# Spatiotemporal characterization of ghrelin and cholecystokinin levels in the gastrointestinal tract of juvenile S*parus aurata*: effects of feeding status and diet composition

**DOI:** 10.3389/fendo.2026.1734169

**Published:** 2026-01-27

**Authors:** Anyell Caderno, Patrik Tang, Verónica de las Heras, Naouel Gharbi, Francisco Javier Alarcón-López, Juan Miguel Mancera, Juan Antonio Martos-Sitcha, Neda Gilannejad

**Affiliations:** 1 Department of Biology, Faculty of Marine and Environmental Sciences, Instituto Universitario de Investigación Marina (INMAR), University of Cádiz, Puerto Real, Spain; 2 NORCE Research AS, Climate & Environment Department, Fish Biology and Aquaculture (FBA) Group, Bergen, Norway; 3 Department of Biology and Geology, Faculty of Experimental Sciences, University of Almería, Almería, Spain; 4 LifeBioencapsulation S.L., Parque Científico PITA, Almería, Spain

**Keywords:** appetite regulation, endocrine pathways, fasting, feed intake, gilthead seabream, hormone levels, refeeding, satiety

## Abstract

Appetite regulation in fish relies on complex neuroendocrine pathways within the brain-gut axis, with ghrelin (Ghrl) and cholecystokinin (Cck) as central players. However, their spatial distribution along the gastrointestinal tract (GIT) and responses to feeding status remain poorly understood in teleosts. This study investigated: i) the baseline distribution of Ghrl and Cck levels along the GIT of juvenile *Sparus aurata* fed a commercial diet; ii) their temporal dynamics during short-term fasting and refeeding; and iii) the influence of diet composition on their spatiotemporal profiles. Juveniles were fed for 92 days with: i) a control diet containing 20% fishmeal (CT); ii) a plant protein diet replacing 60% of fishmeal with hydrolyzed plant protein (PP); and iii) the PP diet supplemented with 2% *LB-Green_Grape_* functional additive (GG). Fish were sampled at 2, 6, and 24 h post-feeding (Cf), after 7 days of fasting (Ft), and at 2, 6, and 24 h post-refeeding (Rf). Hormone levels were quantified across five GIT segments, including the stomach (S1) and four equal intestinal segments (S2–S5). Baseline characterization revealed elevated Ghrl content in S3 and S5, whereas Cck levels were highest in S5. During fasting, Ghrl levels declined, while Cck increased in S1, S2, and S5 with distinct temporal patterns. After refeeding, gastric Ghrl levels (S1) decreased within 24 h, potentially reflecting secretion into plasma and involvement in hunger signaling, although plasma levels were not measured. In contrast, Cck levels in the anterior intestine (S2) rose sharply 24 h after refeeding, suggesting an anticipatory response to refeeding, possibly related to a dual role involving both rapid satiety signaling and preparatory modulation of digestive activity. The PP and GG diets maintained high gastric Ghrl (S1) and lowered intestinal Cck (S2) levels after feeding, especially in the PP diet. This pattern may either prolong satiety and reduce feed intake or reflect changes in hormone release due to lower caloric intake, with the PP diet lowering growth and feed efficiency, partially offset by the functional additive. The study maps Ghrl and Cck in the *S. aurata* GIT, showing spatial, temporal, and dietary regulation, with implications for aquaculture nutrition.

## 1 Introduction

Feed intake (FI) is influenced by a balance between three states: hunger, appetite, and fullness (1). Hunger is the body’s physical need for food, which strongly drives the search for and consumption of food. Appetite, or craving, is the desire to eat, often triggered by the food’s appearance, smell, or taste. Fullness, or satiation, is the feeling of being satisfied that follows eating (2). Regulation of these processes and overall energy balance is essential for ensuring health, growth, reproduction, and survival during periods of food scarcity, as well as enabling daily behaviors such as foraging (3). In aquaculture, a deeper understanding of these regulatory systems is critical for developing feeding strategies that are both efficient and sustainable. Since appetite is influenced by both internal signals and external environmental conditions, adjusting feed management accordingly can enhance production efficiency, reduce waste, and improve animal welfare (4).

In fish, as in other vertebrates, appetite regulation depends on complex interactions between the brain and endocrine signals that either stimulate (orexigenic) or suppress (anorexigenic) FI (5). Many of these hormones are produced along the gastrointestinal tract (GIT), from the stomach to the large intestine, in response to food ingestion, contributing to satiety and influencing gut motility and hormonal secretion, with variations depending on the hormone and species (2). Hormonal activity in the GIT plays a central role in maintaining energy homeostasis, which requires a balance between energy intake and expenditure (6, 7). This regulation includes short-term mechanisms, such as signals from gastric or intestinal distension and food’s chemical properties that are transmitted to the hypothalamus to induce satiety (8), as well as long-term mechanisms that adjust feeding behavior based on energy stores and food availability (9). In contrast, hunger is triggered by anticipatory mechanisms involving both central pathways and peripheral hormones that signal energy deficiency and promote food-seeking behavior (10).

Among the short-term peripheral signals, ghrelin (Ghrl) plays a central role in regulating appetite. In teleosts, Ghrl is primarily produced in the stomach or the intestine in stomachless species (5). Its effect is predominantly orexigenic, although some exceptions have been reported in specific fish species. In addition to its direct role in stimulating appetite, Ghrl may also exert indirect effects on FI by enhancing digestive activity, which could influence the initiation of feeding (10). Experimental studies in various fish species have demonstrated that Ghrl administration increases FI and stimulates foraging behavior (11). Beyond its well-established function in appetite regulation, Ghrl is involved in a variety of physiological and metabolic processes, many of which appear to be species-dependent. It acts directly on somatotrophs to stimulate growth hormone (Gh) release and has also been shown to influence water intake in fish (12). Furthermore, Ghrl modulates insulin synthesis and contributes to carbohydrate and glycogen metabolism, as well as to peripheral lipid metabolism (3, 13).

By contrast, cholecystokinin (Cck) is a peptide structurally related to gastrin, which, like Ghrl, is primarily secreted in the GIT but also in the brain (14). This hormone acts mainly as a short-term satiety factor while simultaneously promoting digestion through various actions within the digestive system (10). In teleost fish, Cck plays multiple roles related to feeding and digestion, including the regulation of pancreatic enzyme release, gallbladder contraction, intestinal motility, and FI (11). Administration of Cck has been shown to significantly reduce FI in fish, confirming its role as an appetite inhibitor (7). Its release is triggered by the presence of dietary fats and small peptides, and part of its anorexigenic effect may be due to delayed gastric emptying. Furthermore, Cck may interact synergistically with long-term adiposity signals such as leptin and insulin in both mammals and fish (9).

Feed deprivation and refeeding experimental setups are widely used to investigate the regulation of appetite and FI in fish (5). However, most studies rely on molecular markers of the brain-gut axis rather than measuring the hormone concentrations (15–18). Analyses of appetite regulation can be particularly relevant for testing novel feed ingredients, as many have been shown to suppress growth primarily through a direct reduction in palatability and FI (19). These experimental settings therefore offer a controlled way to assess how these ingredients affect endocrine signaling and the recovery of essential physiological processes, such as digestion. While the central control of FI has been recently investigated in some aquaculture species to assess novel feed ingredients (20–22), these studies are mainly limited to mRNA-level analyses.

In gilthead seabream (*Sparus aurata*), a species of high aquaculture value in the Mediterranean region, some studies have examined the expression of *ghrl* and *cck* genes along the GIT. Findings indicate that gene expression patterns for both hormones are influenced by feeding frequency (23), fasting (24), and nutritional status and diet composition (25, 26). A study conducted on *Solea senegalensis* measured intestinal Cck hormone levels in the GIT and examined the effects of diurnal versus nocturnal feeding on their daily rhythms under different feeding protocols (27). However, despite these advances, studies addressing how Ghrl and Cck levels are distributed and regulated along the GIT, particularly under varying physiological conditions, remain scarce. To address this gap, the present study aimed i) to provide a detailed characterization of Ghrl and Cck concentrations along the GIT in juvenile *S. aurata*; ii) to examine how short-term feed deprivation and subsequent refeeding influence the spatiotemporal dynamics of these hormone levels; and iii) to assess the impact of dietary composition on the dynamics of both hormones. Although other neuropeptides and hormones are also involved in appetite regulation in teleosts, only Ghrl and Cck were selected to focus on two of the main peripheral signals influencing feeding and digestive processes in mammals and fish (8–11), allowing a detailed assessment of their spatiotemporal dynamics facing different nutritional challenges. To our knowledge, this is the first study to comprehensively quantify and map the distribution of Ghrl and Cck along the GIT in *S. aurata* by measuring hormone levels.

## 2 Materials and methods

### 2.1 Ethics

Fish were handled and maintained in accordance with the guidelines for experimental procedures involving animals, as established by the Ethics and Animal Welfare Committee of the University of Cádiz, as well as by Spanish regulations (RD 53/2013 and RD 118/2021) and European Union legislation (Directive 2010/63/EU). The experimental protocol was approved by the Ethics Committee of the Andalusian Regional Government (Junta de Andalucía, reference number 23/10/2019/176).

### 2.2 Experimental diets and rearing conditions

The experiment was conducted from September to December 2023 at the indoor facilities of the *Servicios Centrales de Investigación en Cultivos Marinos* (SCI-CM, Spanish Operational Code REGA ESII0280003I2) of the University of Cádiz (CASEM, Puerto Real, Cádiz, Spain). After an initial acclimation period of 14 days, *S. aurata* juveniles, with an initial mean weight of 13.33 ± 0.16 g, were randomly distributed in nine 400 L tanks, corresponding to an initial stocking density of ~1.6 kg/m³ (n = three tanks per experimental group; n = 30 fish per tank; n = 90 fish per experimental group), with eight water renewals per day. The fish were maintained under stable conditions, with a temperature ranging from 19°C to 20°C, oxygen saturation greater than 85%, a salinity of 37 ppt, and a natural photoperiod (10L:14D). Water quality parameters related to nitrogen compounds from excretion were monitored twice weekly, remaining stable and below toxic concentrations throughout the experimental period.

Three isonitrogenous (47% dry weight basis) and isolipidic (18% dry weight basis) diets (**Table 1**) were formulated by the CEIMAR-University of Almería facilities (*Servicio de Piensos Experimentales*, Almería, Spain; https://www.ual.es/universidad/serviciosgenerales/stecnicos/perifericos-convenio/piensos-experimentales) to evaluate the effects of dietary composition on gut hormone dynamics: i) CT: control diet, similar to commercial diets, including 20% of fishmeal (FM); ii) PP: experimental diet in which 60% of FM was replaced by hydrolyzed plant proteins, designed to test potential changes in appetite-regulating signals; and iii) GG: the PP diet supplemented with 2% *LB-Green_Grape_*, a functional ingredient derived from Albariño grape marc extract (ACA NeoGiant extract, I-grape S.L. Santiago de Compostela, Spain; https://i-grape.es) combined with a blend of enzymatically hydrolyzed marine and freshwater microalgae (primarily *Arthrospira* sp., *Chlorella* sp., and *Nannochloropsis* sp.; LifeBioencapsulation S.L., Almería, Spain; https://lifebioencapsulation.com), included to assess whether supplementation could modulate any PP-induced effects. Information on the functional ingredient, including its proximate composition and main polyphenols, is presented in **Table 2**. Fish were fed to visual satiety (*ad libitum*) six days per week for 92 days. To minimize potential bias, the trial was conducted in a blinded manner, with the person responsible for animal care unaware of the diet compositions. FI was recorded weekly.

**Table 1:** Ingredients and proximate composition (% dry matter) of the experimental diets.

Ingredients	Experimental diets		
	CT	PP	GG
Fishmeal LT94^1^	20.00	8.00	8.00
Lysine^2^	1.20	1.20	1.20
Methionine^3^	0.50	0.80	0.80
Squid meal^4^	2.00	2.00	2.00
CPSP90^5^	1.00	1.00	1.00
Krill meal^6^	2.00	2.00	2.00
Wheat gluten^7^	10.00	12.00	12.00
Soybean protein concentrate^8^	22.00	29.20	29.20
Pea protein concentrate^9^	7.50	10.60	10.60
Fish oil^10^	9.00	9.00	9.00
Plant oils^11^	4.30	5.70	5.70
Soybean lecithin^12^	1.00	1.00	1.00
Wheat meal^13^	16.90	14.40	12.40
Monoammonium phosphate^14^	0.50	1.00	1.00
Vitamin and mineral premix^15^	2.00	2.00	2.00
Vitamin C^16^	0.10	0.10	0.10
*LB-Green_Grape_* ^17^	–	–	2.00
Crude protein	46.90	46.40	46.60
Crude lipid	18.20	17.90	18.10
Ash	6.90	5.80	5.80
Nfe^18^	22.70	24.10	23.20
Moisture	5.40	5.80	6.30

Dietary codes: CT: control (fishmeal-based) diet; PP: plant-based diet; GG: plant-based diet supplemented with *LB-Green_Grape_*. ^1^69.4% crude protein, 12.3% crude lipid (Norsildemel, Bergen, Norway). ^2,3^Lorca Nutrición Animal S.A. (Murcia, Spain). ^4,5,6^purchased from Bacarel (UK). CPSP90 is enzymatically pre-digested fishmeal. ^7^78% crude protein (Lorca Nutrición Animal S.A., Murcia, Spain). ^8^Soycomil, 60% crude protein, 1.5% crude lipid (ADM, Poland). ^9^Pea protein concentrate, 85% crude protein, 1.5% crude lipid (Emilio Peña S.A., Spain). ^10^AF117DHA (Afamsa, Spain). ^11^Blend of soybean, rapeseed, and linseed (4:4:2) oils (Aceites el Niño, Spain). ^12^P700IP (Lecico, DE). ^13^Local providers (Almería, Spain). ^14^Lorca Nutrición Animal S.A. (Murcia, Spain). ^15^Lifebioencapsulation S.L. (Almería, Spain). Vitamins (mg kg^-1^): vitamin A (retinyl acetate), 2,000,000 IU; vitamin D3 (DL-cholecalciferol), 200,000 IU; vitamin E (Lutavit E50), 10,000 mg; vitamin K3 (menadione sodium bisulphite), 2,500 mg; vitamin B1 (thiamine hydrochloride), 3,000 mg; vitamin B2 (riboflavin), 3,000 mg; calcium pantothenate, 10,000 mg; nicotinic acid, 20,000 mg; vitamin B6 (pyridoxine hydrochloride), 2,000 mg; vitamin B9 (folic acid), 1,500 mg; vitamin B12 (cyanocobalamin), 10 mg; vitamin H (biotin), 300 mg; inositol, 50,000 mg; betaine (Betafin S1), 50,000 mg. Minerals (mg kg^-1^): Co (cobalt carbonate), 65 mg; Cu (cupric sulphate), 900 mg; Fe (iron sulphate), 600 mg; I (potassium iodide), 50 mg; Mn (manganese oxide), 960 mg; Se (sodium selenite), 1 mg; Zn (zinc sulphate), 750 mg; Ca (calcium carbonate), 18.6% (186,000 mg); KCl, 2.41% (24,100 mg); NaCl, 4.0% (40,000 mg). ^16^TECNOVIT (Spain). ^17^*LB-Green_Grape_* is a fine dry powder composed of a freeze-dried extract from Albariño white grape marc combined with a blend of enzymatically hydrolyzed marine and freshwater microalgae (*Arthrospira platensis, Chlorella vulgaris*, and *Nannochloropsis gaditana*) containing 37,500 ppm of total polyphenols/kg. This product has been developed within the NeoGiant project (Grant 101036768) by I-Grape S.L. and LifeBioencapsulation S.L., Spin-offs from Universidad de Santiago de Compostela and Universidad de Almería, respectively. ^18^Carbohydrate content estimated by calculation as Nitrogen-free extract = 100 - (% crude protein + % crude lipid + % ash).

**Table 2:** Chemical composition (% dry matter) and polyphenol profile ( mg·kg^-1^) of the functional ingredient *LB-Green_Grape_*. .

Proximate composition (%)	
Crude protein	11.50 ± 0.90
Crude lipid	2.00 ± 0.70
Total carbohydrates	83.40 ± 1.40
Ash	3.10 ± 0.20
Main polyphenols (mg/kg)	
Gallic acid	239.20 ± 15.70
Caftaric acid	15.90 ± 2.10
Procyanidins (B1+B2+C1)	1681.50 ± 51.30
Catechin	1813.70 ± 88.30
Epicatechin	1201.30 ± 59.80
Epicatechin gallate	78.40 ± 6.30
Quercetin-3-rutinoside	11.00 ± 2.30
Quercetin-3-glucoside	264.80 ± 12.10
Quercetin-3-glucuronide	283.20 ± 15.20
Quercetin	22.50 ± 3.10
Total phenolic index (mg GAE/kg)^1^	35607.60 ± 534.10

^1^GAE, Gallic Acid Equivalents.

### 2.3 Experimental design and sampling procedures

**Figure 1:**
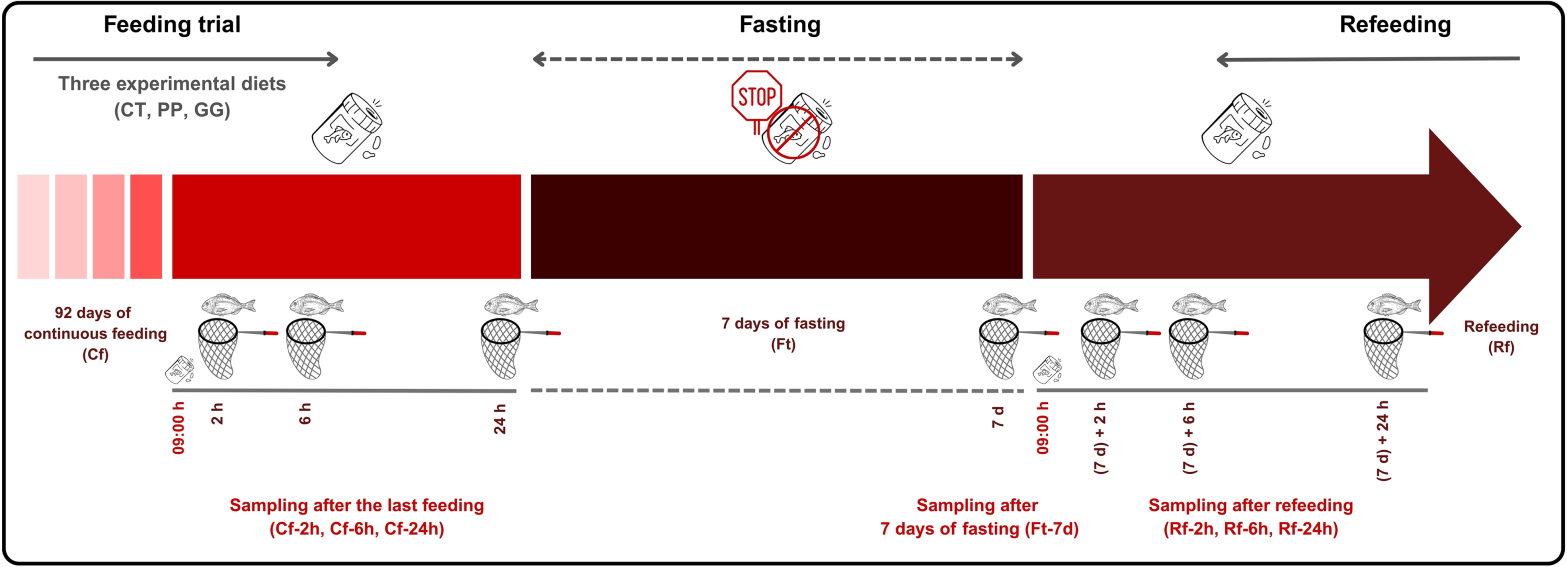
Detailed schematic representation of the experimental design conducted with juvenile *Sparus aurata* fed continuously to apparent visual satiety for 92 days using three experimental diets (CT: control diet based on 20% FM; PP: diet with 60% replacement of FM by hydrolyzed plant protein; GG: PP diet supplemented with 2% of the *LB-Green_Grape_*). After the continuous feeding period (Cf), fish underwent a 7-day fasting phase (Ft) followed by refeeding (Rf). The timeline indicates the seven sampling points (solid line: feeding and refeeding phases; dashed line: fasting phase).

At the end of the experimental period (**Figure 1**), fish were sampled at 2, 6, and 24 h after the last feed at 09:00 (referred to as Cf-2h, Cf-6h, and Cf-24h, respectively), under continuous feeding conditions (Cf). Following this, the fish underwent an additional six days of fasting (Ft), completing a total of seven consecutive days without feeding, and were sampled again at the end of this period (Ft-7d). Finally, the fish were refed at 09:00 (Rf) with their respective experimental diets and sampled once more at 2, 6, and 24 h after refeeding (Rf-2h, Rf-6h, and Rf-24h). At each sampling point, 6–7 specimens per experimental diet (across the three tanks) were randomly selected and deeply anesthetized using a lethal dose of 2-phenoxyethanol (1 mL/L seawater). The stomach and intestine of each fish were carefully removed and separated, with the pyloric caeca left attached to the intestine. Samples were then snap-frozen in liquid nitrogen and stored at −80°C.

The following zootechnical parameters were evaluated: i) Weight gain (WG/%) = 100 × (body weight increase/initial body weight); ii) Specific Growth Rate (SGR, %/day) = 100 × [(ln final body weight − ln initial body weight)/number of days]; and iii) Feed Conversion Ratio (FCR) = total feed intake/weight gain.

### 2.4 Hormone characterization and analysis

#### 2.4.1 Experimental phases

**Figure 2:**
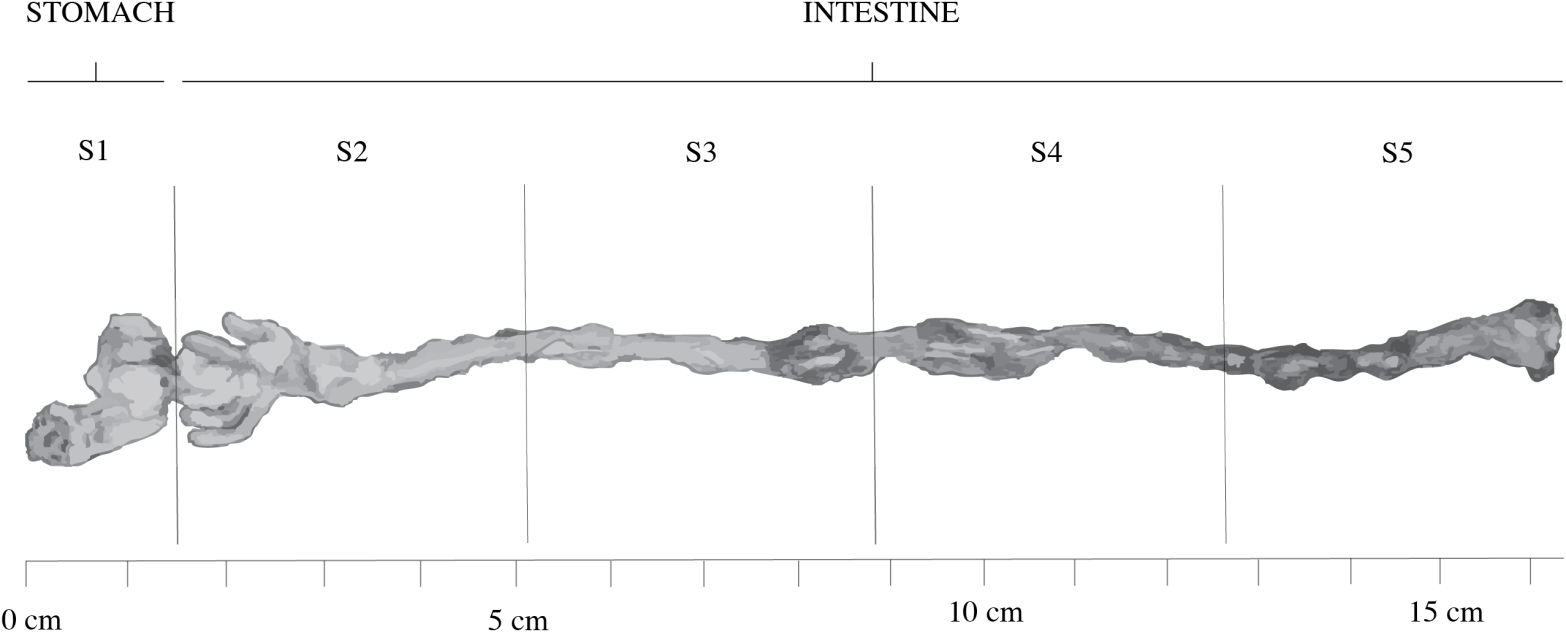
Anatomical diagram of the gastrointestinal tract (GIT) of juvenile *Sparus aurata*, divided into five segments (S): S1, stomach; S2, pyloric caeca and proximal half of the anterior intestine; S3, distal half of the anterior intestine; S4, proximal half of the posterior intestine; and S5, distal half of the posterior intestine, including the rectum.

The GIT was divided into five regions (**Figure 2**) to ensure anatomically consistent and unbiased sampling across individuals. The stomach was treated as a distinct unit (S1), while the intestine was subdivided into four equal-length segments (S2–S5). The intestinal tract of *S. aurata* is commonly described as having anterior, mid, and posterior regions (28). However, these boundaries are difficult to distinguish macroscopically during dissection, as morphological transitions are gradual and become clearer at the histological level. Therefore, proportional segmentation provided a reproducible operational framework and avoided subjective assignment of regional limits. The pyloric caeca were included within segment S2.

For a comprehensive characterization and analysis of Ghrl and Cck throughout the GIT in *S. aurata*, the experiment was organized into three distinct phases:

##### 2.4.1.1 Phase 1

A preliminary regional characterization of Ghrl and Cck content along the GIT was conducted using seven juvenile gilthead seabream (mean weight: 10.10 ± 0.12 g) that were not included in the feeding trial. These fish were acclimated in our facilities, fed a commercial diet to visual satiety six days per week (not the CT, PP, or GG experimental diets), and thus maintained under baseline physiological conditions without exposure to nutritional or experimental challenges. GIT samples were collected 4 h after the last feeding (09:00) to determine the distribution and concentration of each hormone across the five GIT segments.

##### 2.4.1.2 Phase 2

To evaluate the temporal dynamics of both hormones, Ghrl and Cck levels were measured at four initial sampling points (Cf-2h, Cf-6h, Cf-24h, and Ft-7d) in fish that were fed only the CT diet (*ad libitum* six days per week, as described above). Analyses were performed in segments S1, S2, and S5.

##### 2.4.1.3 Phase 3

To analyze how diet composition influences Ghrl and Cck levels and their temporal patterns, fish fed the three experimental diets (CT, PP, and GG) were sampled across the full time course (Cf-2h, Cf-6h, Cf-24h, Ft-7d, Rf-2h, Rf-6h, and Rf-24h). In this phase, Ghrl was quantified only in the stomach (S1) and Cck only in the proximal intestine (S2).

The objective of Phase 1 was to provide a basic, initial map of the regionalization of both hormones throughout the GIT of *S. aurata* (baseline characterization). Segment selection for Phases 2 and 3 followed functional criteria grounded in teleost physiology and the distribution patterns observed in Phase 1. The stomach (S1) was chosen for Ghrl measurements because, in species with a functional stomach, it is a major site of Ghrl production during feeding–fasting–refeeding transitions. The proximal intestine (S2), including the pyloric caeca, was selected for Cck quantification, given its believed involvement in early digestive processing and satiety signaling (3, 29, 30). The distal intestine (S5) was included in Phase 2 temporal analyses due to unexpectedly high hormone levels in that region, allowing us to test whether these distal elevations reflected transient post-feeding dynamics. In contrast, Phase 3 focused exclusively on S1 for Ghrl and S2 for Cck to evaluate dietary modulation, due to clear endocrine roles, regulatory functions, and direct functional relevance to the research questions in this study.

#### 2.4.2 Tissue processing

Tissue processing followed the procedure described previously in (27), with slight modifications. For hormone quantification, each GIT segment was opened and thoroughly rinsed using forceps in ice-cold 1× Phosphate-Buffered Saline (PBS; Sigma-Aldrich, P5493) to remove residual feed and digesta.

Cleaned tissue samples were transferred into pre-weighed 5 mL tubes containing 1× PBS (prepared from a 10× stock solution). The tissue was then homogenized at a 1:9 (w/v) ratio using the PBS solution with an Ultra-Turrax^®^. Homogenates were centrifuged in two consecutive batches at 4°C and 1500 × g for 15 minutes. Resulting supernatants were aliquoted and stored at −20°C until analysis. The homogenization protocol was chosen after confirming that hormone concentrations were comparable between samples processed as whole tissue (as in this study) and those prepared by scraping the epithelial mucosa layer, as initially described in (27). The selected method enabled greater efficiency in processing many samples without compromising the reliability of the measurements.

#### 2.4.3 Hormone quantification

Ghrl concentration was quantified using a sandwich Fish Ghrelin ELISA Kit (Ref. No. MBS1601713; MyBioSource, USA). Cholecystokinin concentration was quantified using a sandwich Fish Cholecystokinin-8 ELISA Kit (Ref. No. MBS069488; MyBioSource, USA). In both cases, the protocol was performed according to the manufacturer’s instructions. In brief, for each hormone, tissue extracts and standards were added to antibody-coated microplates, incubated with the corresponding detection antibodies and enzyme conjugates, and then washed to remove unbound material. Color was developed using a chromogenic substrate, the reaction was stopped, and absorbance was read at 450 nm. Samples were assayed in duplicate, and their concentrations extrapolated from a standard curve (15–240 ng·L^–1^ for Ghrl, and 15.6–500 pg·mL^−1^ for Cck) and expressed as ‘ng per g of wet weight (ng·g^-1^ ww)’, considering the total amount of tissue used for hormone determination.

Theoretical validation of these kits for *S. aurata* is presented in the **Supplementary Material**. Briefly, due to limited sequence information and annotation in this species, the potential detection of multiple Cck isoforms (Cck-8, Cck-10, Cck-33) cannot be excluded; therefore, the generic term ‘Cck’ is used throughout the manuscript. Alignment of the antibody epitopes with the available *S. aurata* sequences indicated that the Cck ELISA kit most likely detects Cck-like immunoreactivity predominantly derived from the *cckb* gene in the gastrointestinal tract. For Ghrl, the lower sequence identity between the kit antibodies and the *S. aurata* Ghrl sequence may reduce binding efficiency; nevertheless, the assay remains suitable for comparing relative differences in Ghrl-like immunoreactivity among gastrointestinal segments, as performed in the present study. Additionally, potential cross-reactivity with gastrin (for Cck) and motilin (for Ghrl) cannot be fully excluded. Therefore, throughout this manuscript, the terms ‘Cck’ and ‘Ghrl’, when referring to measurements from the present study, refer to Cck-like and Ghrl-like immunoreactivity, respectively, reflecting the theoretical and methodological limitations described above.

### 2.5 Statistical analysis

All results are presented as mean ± standard error of the mean (SEM). Prior to statistical analysis, data normality and homogeneity of variances were assessed using the Kolmogorov–Smirnov and Levene’s tests, respectively. A one-way analysis of variance (ANOVA) was performed separately to: i) compare differences among GIT segments (S1–S5) during Phase 1 (initial characterization) and in Phase 2 (for validation of Phase 1); ii) evaluate Ghrl and Cck levels in segments S1, S2, and S5 across experimental diets (CT, PP, GG) during the first 24 h post-feeding (Phase 3); and iii) assess growth performance and feed utilization parameters for each experimental diet. Subsequently, separate two-way ANOVAs were conducted, each including sampling time points (Cf-2h, Cf-6h, Cf-24h, Ft-7d, Rf-2h, Rf-6h, Rf-24h) and one of the following fixed factors: hormone type, GIT segments, or experimental diets. When significant effects were found (*p < 0.05*), Tukey’s *post hoc* test was used for pairwise comparisons. Correlation analyses between Ghrl and Cck levels were performed separately within three physiological states and independently for each experimental diet: i) Postprandial state (Cf-2h, Cf-6h, Cf-24h); ii) fasting state (Ft-7d); and iii) refeeding state (Rf-2h, Rf-6h, Rf-24h). Pearson’s correlation coefficients (r) were used when normality assumptions were met, and Spearman’s rank (ρ) correlations were applied otherwise. Statistical significance was defined as follows: *p < 0.05* (*), *p < 0.01* (**), *p < 0.001* (***), and *p < 0.0001* (****). All statistical analyses and graphical representations were performed using GraphPad Prism version 8.0 (GraphPad Software, Inc., San Diego, CA, USA).

## 3 Results

### 3.1 Phase 1. Baseline characterization of appetite hormone levels across the GIT

**Figure 3:**
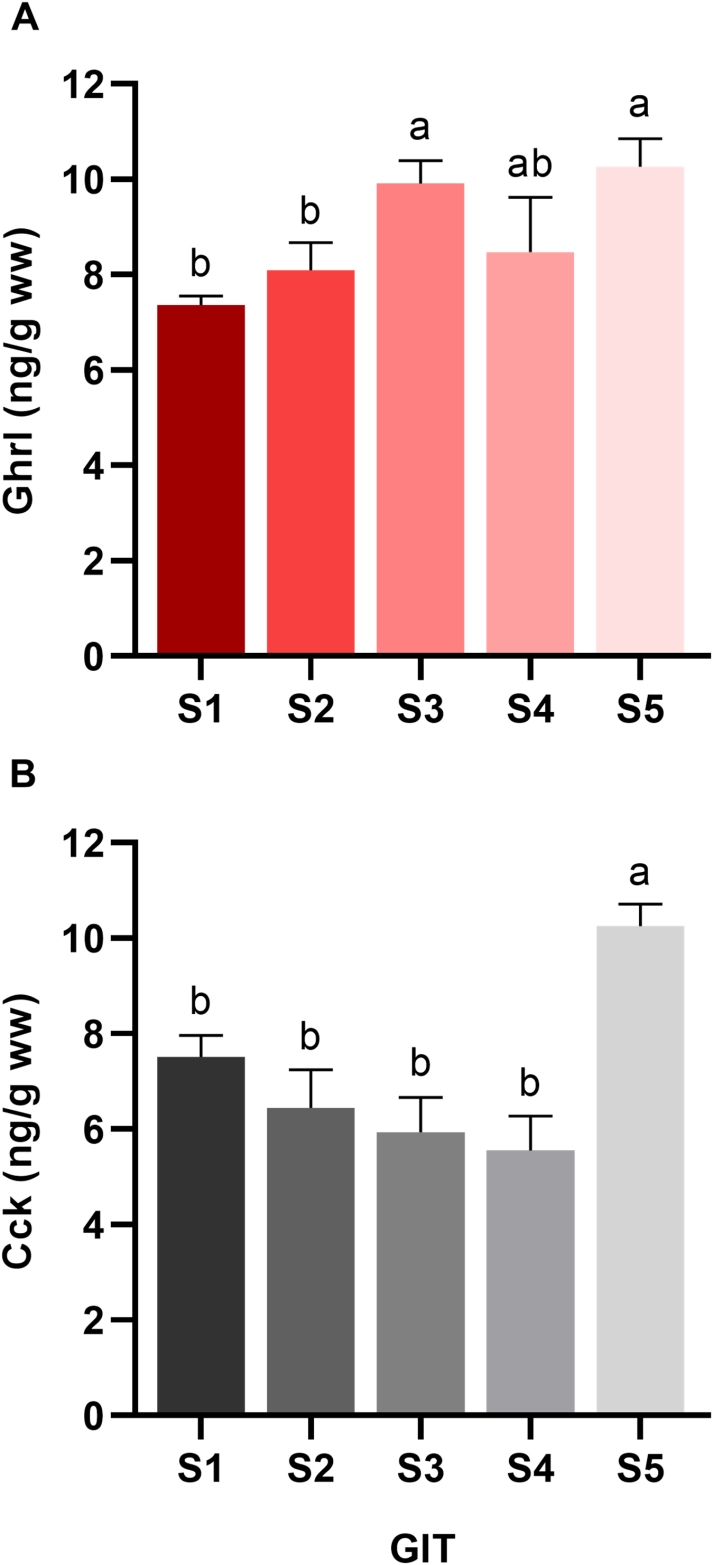
Baseline characterization of **(A)** ghrelin (Ghrl) and **(B)** cholecystokinin (Cck) levels across five gastrointestinal segments (S) in juvenile *Sparus aurata* not subjected to any nutritional challenge and fed a commercial diet. Fish were fed at 09:00 h, and samples were collected 4 h after feeding. Hormone concentrations are expressed as ng·g^-1^ wet weight. Data are presented as mean ± SEM (n = 7 fish). Different letters indicate statistically significant differences among segments, as determined by one-way ANOVA followed by Tukey’s *post hoc* test (*p < 0.05*).

Juvenile *S. aurata* fed a commercial diet, not subjected to any experimental challenge, were sampled 4 h after their last feeding (09:00 h). Ghrl levels were significantly higher in S3 and S5 of the GIT compared with S1 and S2 (*p < 0.05*), while S4 did not differ significantly from the other segments (**Figure 3A**). Regarding Cck values (**Figure 3B**), a decreasing trend from S1 to S4 was observed, although not significant (*p > 0.05*). Nonetheless, Cck levels increased notably in S5 (*p < 0.05*).

### 3.2 Temporal fluctuations of Ghrl and Cck levels after feeding and during fasting

**Figure 4:**
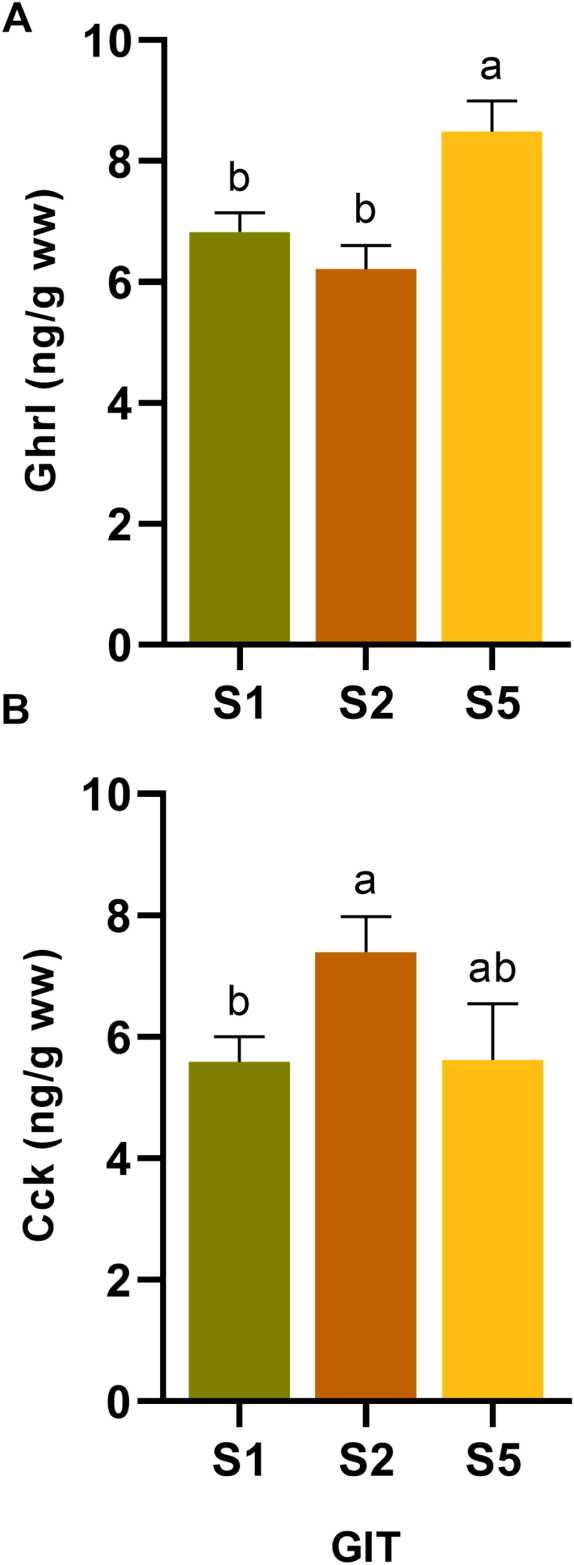
Mean values of **(A)** ghrelin (Ghrl) and **(B)** cholecystokinin (Cck) during the first 24 h after the last feeding (09:00; average of Cf-2h, Cf-6h, and Cf-24h) in segments S1, S2, and S5 of the GIT of juvenile *Sparus aurata* fed to apparent visual satiety for 92 days with the control diet (CT: control diet based on 20% FM). Hormone concentrations are expressed as ng·g^-1^ wet weight. Data are presented as mean ± SEM (n = 18 fish per time point, randomly sampled across three replicate tanks). Different letters indicate statistically significant differences among segments, as determined by one-way ANOVA followed by Tukey’s *post hoc* test (*p < 0.05*).

To validate the results obtained in Phase 1, fish fed the control diet (CT) during the feed trial were used to compare total hormone values over the first 24 h, calculated as the average of hormone levels at 2, 6, and 24 h post-feeding, in segments S1, S2, and S5. For Ghrl, a similar content distribution pattern was observed (**Figure 4A**), with significantly higher levels in S5 compared with S1 and S2 (*p < 0.05*). In contrast, Cck levels varied among segments (**Figure 4B**), being highest in S2, which differed significantly from S1 (*p < 0.05*) but not from S5 (*p > 0.05*).

**Figure 5:**
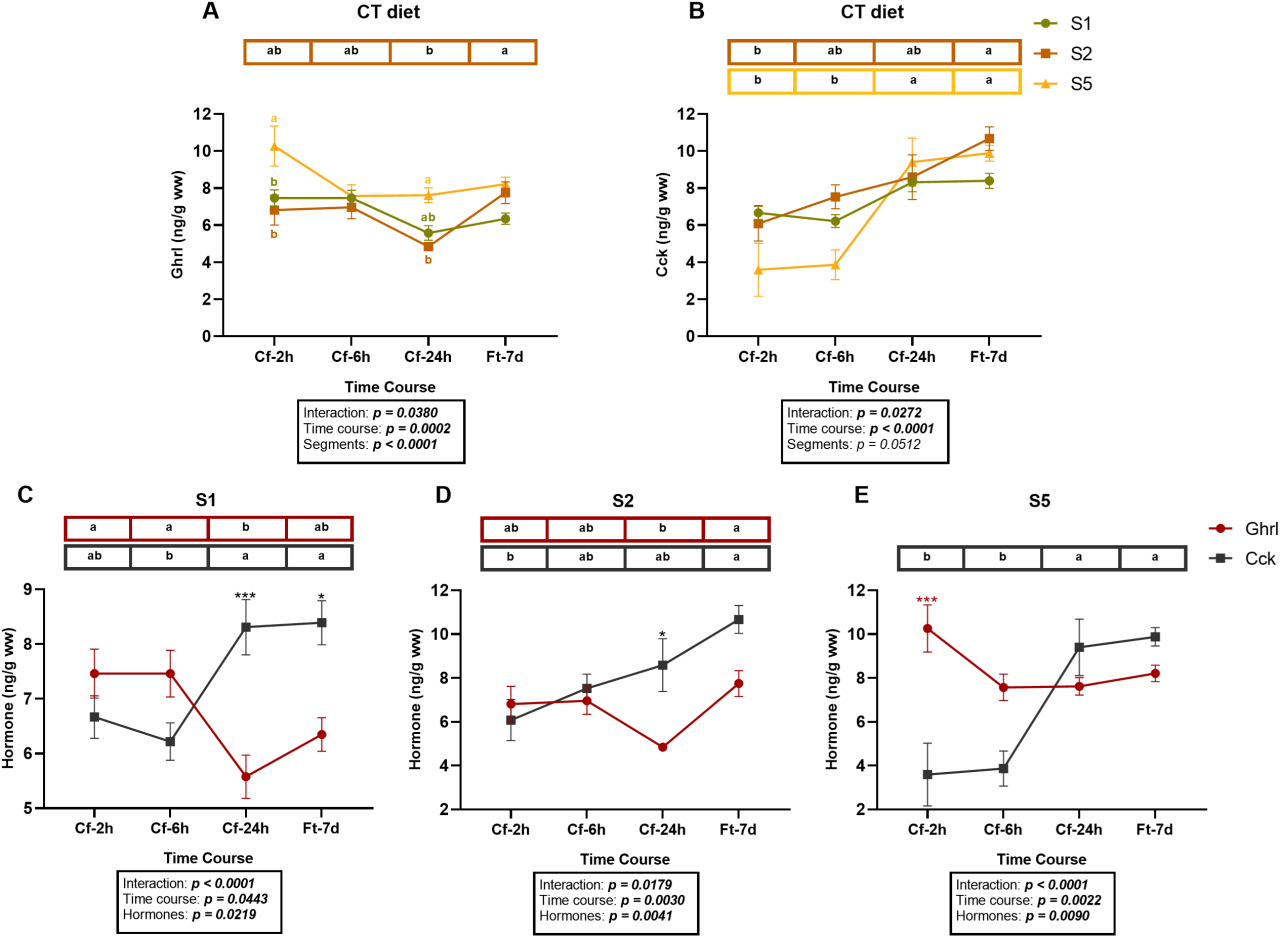
Two graphical representations of the same dataset showing ghrelin (Ghrl) and cholecystokinin (Cck) hormone levels in juvenile *Sparus aurata* fed to apparent visual satiety for 92 days with the control diet (CT: control diet based on 20% FM). **(A)** Ghrl and **(B)** Cck across the GIT segments S1, S2, and S5. The two hormones were analyzed separately in segments **(C)** S1, **(D)** S2, and **(E)** S5. Samples were collected at 2, 6, and 24 h after the last feeding (09:00; Cf-2h, Cf-6h, Cf-24h) and after 7 days of fasting (Ft-7d). Hormone concentrations are expressed as ng·g^-1^ wet weight. Data are presented as mean ± SEM (n = 6 fish per time point, randomly sampled across three replicate tanks). Different black letters indicate statistically significant differences among sampling times within each GIT segment or hormone; colored letters indicate differences between segments at a specific time point; and asterisks indicate differences between Ghrl (red) and Cck (black) at a specific time point, as determined by two-way ANOVA followed by Tukey’s *post hoc* test: *p < 0.05* (*) and *p < 0.001* (***).

Regarding the temporal fluctuations of hormone levels in segments S1, S2, and S5 in juveniles fed CT diet, significant effects of time, segment, and their interaction were observed for Ghrl concentrations (**Figure 5A**). These levels were significantly higher in S5 compared with S1 and S2 at 2- and 24-h post-feeding (Cf-2h and Cf-24h; *p < 0.05*). However, at 24 h post-feeding (Cf-24h), levels in the stomach (S1) were no longer significantly different from those in S2 and S5. Across the time course, significant temporal fluctuations were only detected in S2 (*p < 0.05*), where Ghrl levels markedly decreased at Cf-24h, followed by a significant increase after seven days of fasting (Ft-7d).

In the case of Cck (**Figure 5B**), no significant differences were observed between segments (*p > 0.05*), although a significant effect of time and time × segment interaction was detected. In S1, Cck levels remained stable over time (*p > 0.05*). In contrast, S2 progressively enhanced Cck values with the duration of fasting, reaching a peak at Ft-7d (*p < 0.05*). In S5, the increase was not immediate, but levels were significantly elevated at both Cf-24h and Ft-7d compared to 2- and 6-h post-feeding (Cf-2h and Cf-6h; *p < 0.05*).

When temporal variations of both hormones were analyzed simultaneously in each segment (**Figures 5C–E**), a complementary pattern emerged. A two-way ANOVA revealed significant effects of hormone (*p < 0.05*), time (*p < 0.05*), and their interaction (hormone × time, *p < 0.05*), justifying a temporal analysis within each segment.

In the stomach (S1), an inverse pattern between Ghrl and Cck levels was evident (**Figure 5C**). Ghrl concentrations were higher at Cf-2h and Cf-6h (*p < 0.05*). However, the difference between Ghrl and Cck content at these time points was not statistically significant (*p > 0.05*). As the fasting period progressed, a shift occurred, with Cck levels becoming significantly higher than Ghrl at Cf-24h and Ft-7d (*p < 0.001* and *p < 0.05*, respectively). These statistical differences in S1 were not detected in the individual hormone analyses in each segment (**Figures 5A, B**, green line), likely due to greater hormonal fluctuations in S2 and S5. Importantly, the same dataset was used for all analyses; therefore, differences in statistical outcomes are attributable solely to the comparative approach.

In S2, the pattern diverged from that observed in S1 (**Figure 5D**), with an early reversal in hormone levels between Cf-2h and Cf-6h. Cck levels progressively increased, surpassing Ghrl levels at Cf-6h, Cf-24h, and Ft-7d, although the difference was only statistically significant at Cf-24h (*p < 0.05*). Ghrl concentrations decreased to a minimum at Cf-24h and subsequently increased significantly after seven days of fasting (Ft-7d; *p < 0.05*). These temporal variations and statistical outcomes were consistent with those observed in the individual hormone analyses (**Figures 5A, B**, orange line), although the joint analysis allows for clearer visualization of the differences.

In S5, although the overall pattern was similar to S1, some differences were observed (**Figure 5E**). The most pronounced divergence occurred early: Ghrl levels were significantly higher than Cck at Cf-2h (*p < 0.001*). Between Cf-6h and Cf-24h, this pattern reversed, with Cck surpassing Ghrl at Cf-24h and Ft-7d, although the difference was not significant (*p > 0.05*). In this segment, the statistical differences observed in joint analysis matched those seen in **Figures 5A and B** (yellow line), confirming the consistency of the dataset while highlighting the advantage of simultaneous hormone comparisons in detecting subtle temporal dynamics.

### 3.3 Phase 3. Effect of diet composition on hormonal response over time

No mortality was observed in any of the experimental groups throughout the study period. The effects of the experimental diets on growth performance are summarized in **Table 3**. Significant differences were detected in final body weight, WG, and SGR, with the CT group achieving the highest values, the PP group the lowest, and the GG group presenting intermediate values (*p < 0.05*). FCR also differed significantly among treatments; the PP group exhibited the highest FCR, whereas the CT and GG groups showed similarly lower values (*p < 0.05*).

**Table 3:** Growth performance of juvenile *Sparus aurata* fed to apparent visual satiety for 92 days with three experimental diets (CT: control diet based on 20% FM; PP: diet with 60% replacement of FM by hydrolyzed plant protein; GG: PP diet supplemented with 2% of the *LB-Green_Grape_*).

Parameters	Experimental diets			*p-value*
	CT	PP	GG	
Initial weight (g)	13.32 ± 0.26	13.23 ± 0.04	13.45 ± 0.04	*0.5790*
Final weight (g)	54.22 ± 1.53^a^	48.14 ± 1.15^b^	50.74 ± 1.28^ab^	*0.0069*
WG (%)^1^	294.10 ± 9.61^a^	247.10 ± 7.21^b^	270.40 ± 11.16^ab^	*0.0087*
SGR (%/day)^2^	1.56 ± 0.03^a^	1.41 ± 0.02^b^	1.48 ± 0.02^ab^	*0.0006*
FCR^3^	1.62 ± 0.01^b^	1.77 ± 0.01^a^	1.64 ± 0.03^b^	*<0.0001*

^1^Weight gain (%) = 100 × (body weight increase/initial body weight).

^2^Specific growth rate = 100 × [(ln final body weight – ln initial body weight)/days].

^3^Feed conversion ratio = total feed intake/weight gain.

Growth parameters are presented as mean ± SEM from triplicate tanks. Different superscript letters indicate statistically significant differences between experimental diets, as determined by one-way ANOVA followed by Tukey’s *post hoc* test (*p < 0.05*).

**Figure 6:**
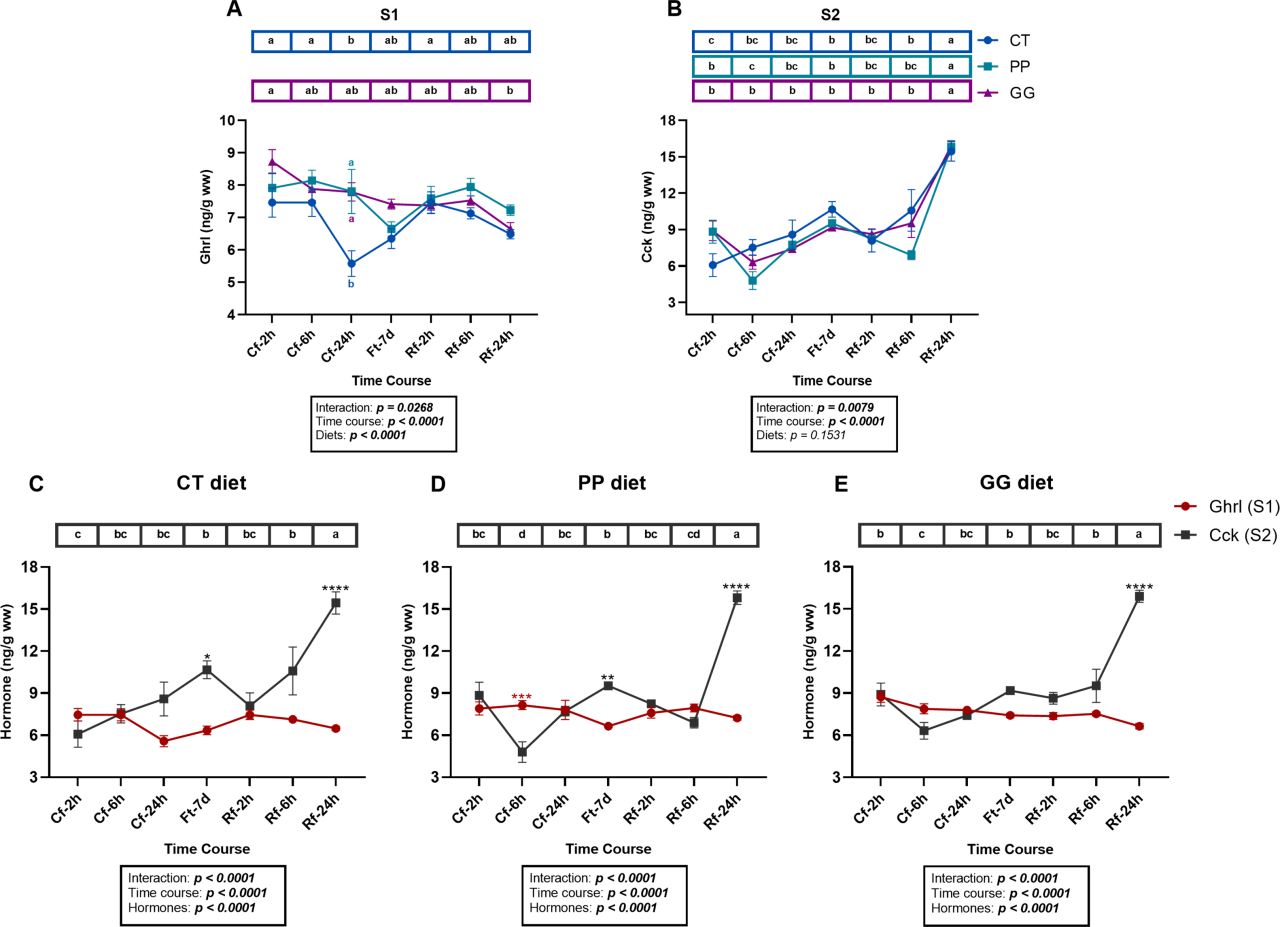
Two graphical representations of the same dataset showing ghrelin (Ghrl) and cholecystokinin (Cck) hormone levels measured in S1 and S2, respectively, in juvenile *Sparus aurata* fed to apparent visual satiety for 92 days with three experimental diets (CT: control diet based on 20% FM; PP: diet with 60% replacement of FM by hydrolyzed plant protein; GG: PP diet supplemented with 2% of the *LB-Green_Grape_*). **(A)** Ghrl, **(B)** Cck. The two hormones were analyzed separately for experimental diets in **(C)** CT diet, **(D)** PP diet, and **(E)** GG diet. Samples were collected at 2, 6, and 24 h after the last feeding (09:00; Cf-2h, Cf-6h, Cf-24h), after 7 days of fasting (Ft-7d), and following refeeding (09:00; Rf-2h, Rf-6h, Rf-24h). Hormone concentrations are expressed as ng·g^-1^ wet weight. Data are presented as mean ± SEM (n = 6–7 fish per time point, randomly sampled across three replicate tanks). Different black letters indicate statistically significant differences among sampling times within each diet or hormone; colored letters indicate differences between experimental diets at a specific time point; and asterisks indicate differences between Ghrl (red) and Cck (black) at a specific time point, as determined by two-way ANOVA followed by Tukey’s *post hoc* test: *p < 0.05* (*), *p < 0.01* (**), *p < 0.001* (***), and *p < 0.0001* (****).

To assess dietary effects on hormone levels, Ghrl was analyzed in S1 and Cck in S2 in fish fed the three experimental diets (CT, PP, and GG), including the refeeding phase (see Section 2.4). A two-way ANOVA revealed a significant interaction between diet and time for both hormones (*p < 0.001*), supporting a diet-specific analysis (**Figure 6**).

For Ghrl (**Figure 6A**), a significant effect of diet was detected (*p < 0.05*), but fish fed the PP diet showed no temporal variation throughout the experimental timeline (*p > 0.05*). In contrast, those fed the CT diet exhibited a significant decrease in Ghrl levels at 24 h post-feeding (Cf-24h; *p < 0.05*), which was also the only time point where significant differences among dietary treatments were observed (*p < 0.05*), with higher Ghrl values in both PP and GG groups compared to CT. In the GG-fed group, although Ghrl levels remained relatively stable, a slight progressive decrease was observed throughout the experimental period, becoming significant 24 h after refeeding (Rf-24h; *p < 0.05*).

Regarding Cck levels (**Figure 6B**), no significant effect of diet was detected (*p > 0.05*), but temporal variation was observed within each diet. In all dietary groups, Cck values peaked at both seven days of fasting (Ft-7d) and 24 h after refeeding (Rf-24h), with the latter increase being particularly pronounced (*p < 0.05*). In fish fed the GG diet, Cck levels remained relatively stable throughout the fasting-refeeding cycle, with a significant increase only at Rf-24h. A tendency toward decreased Cck values was observed at 6 h post-feeding (Cf-6h) in both GG and PP groups, but only statistically significant in PP (*p < 0.05*). In contrast, fish fed the CT diet showed lower Cck concentrations shortly after feeding events (Cf-2h and Rf-2h).

When temporal variations of both hormones were analyzed for each dietary group (**Figures 6C–E**), a complementary perspective emerged. A two-way ANOVA confirmed significant effects of hormone (*p < 0.05*), time (*p < 0.05*), and their interaction (hormone × time, *p < 0.05*). Direct comparison of Cck and Ghrl values across time within each diet allowed a more precise visualization of temporal patterns. While Cck showed clear and significant temporal variations (*p < 0.05*), Ghrl fluctuations were less evident in all three experimental groups (*p > 0.05*). This pattern was likely influenced by the sharp Cck peak observed at the end of the experimental period (Rf-24h), which dominated the combined hormone profile.

In fish fed the CT diet (**Figure 6C**), Cck levels progressively increased over the course of the experiment, with the only noticeable drop occurring at 2 h after refeeding (Rf-2h). These levels remained higher than Ghrl throughout most of the timeline, although significant differences between both hormones were only detected at Ft-7d and Rf-24h (*p < 0.05* and *p < 0.0001*, respectively). In the PP-fed group (**Figure 6D**), Ghrl values were significantly higher at 6 h post-feeding (Cf-6h; *p < 0.001*), while Cck levels peaked significantly at Ft-7d and Rf-24h (*p < 0.01* and *p < 0.0001*, respectively), with the latter being consistent with observations in the CT group. Unlike CT, however, Cck levels showed two significant declines in this group, specifically at 6 h after both feeding events (Cf-6h and Rf-6h; *p < 0.05*). In fish fed the additive-supplemented diet (GG; **Figure 6E**), no significant differences between Ghrl and Cck levels were detected at most time points, except for the distinct Cck peak at Rf-24h (*p < 0.0001*). Similar to the PP group, a decrease in Cck values was noted at 6 h post-feeding (Cf-6h).

**Figure 7:**
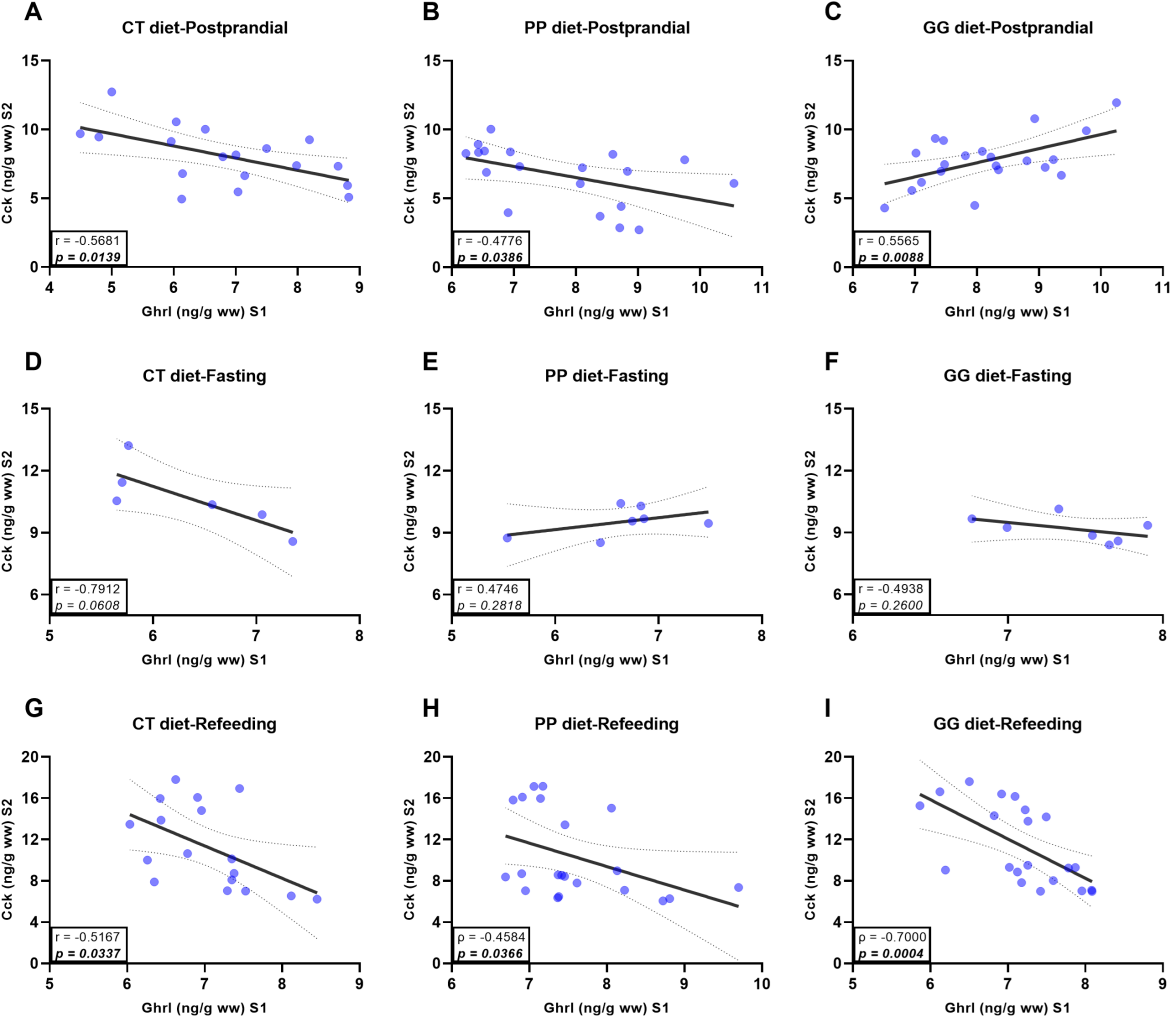
Correlation between gastric ghrelin (Ghrl) and intestinal cholecystokinin (Cck) hormone levels measured in S1 and S2, respectively, in juvenile *Sparus aurata* fed to apparent visual satiety for 92 days with three experimental diets (CT: control diet based on 20% FM; PP: diet with 60% replacement of FM by hydrolyzed plant protein; GG: PP diet supplemented with 2% of the *LB-Green_Grape_*). **(A)** Postprandial state in CT diet, **(B)** Postprandial state in PP diet, **(C)** Postprandial state in GG diet, **(D)** Fasting state in CT diet, **(E)** Fasting state in PP diet, **(F)** Fasting state in GG diet, **(G)** Refeeding state in CT diet, **(H)** Refeeding state in PP diet, and **(I)** Refeeding state in GG diet. Postprandial state: samples were collected at 2, 6, and 24 h after the last feeding (09:00; Cf-2h, Cf-6h, Cf-24h). Fasting state: samples were collected following 7 days of fasting (Ft-7d). Refeeding state: samples were collected after refeeding (09:00; Rf-2h, Rf-6h, Rf-24h). Hormone concentrations are expressed as ng·g^-1^ wet weight. Data are presented as mean ± SEM (n = 6–7 fish per time point, randomly sampled across three replicate tanks). Pearson correlation (r) or Spearman correlation (ρ) coefficients. The dashed lines represent the 95% confidence intervals.

Correlation analyses performed separately for each dietary treatment and feeding condition revealed consistent inverse associations between stomach Ghrl and anterior-intestine Cck in most cases (**Figure 7**). In the postprandial phase (Cf-2h, Cf-6h, and Cf-24h), both the CT (**Figure 7A**) and PP (**Figure 7B**) groups showed significant negative correlations between the two peptides (*p < 0.05*). In contrast, fish fed the GG diet exhibited a significant positive correlation during this same postprandial window (*p < 0.05*; **Figure 7C**). During fasting (Ft-7d), no significant association was detected between gastric Ghrl and anterior-intestine Cck (*p > 0.05*; **Figures 7D–F**). In the refeeding phase (Rf-2h, Rf-6h, and Rf-24h), a significant negative relationship re-emerged across all diets (*p < 0.05*; **Figures 7G–I**).

**Figure 8:**
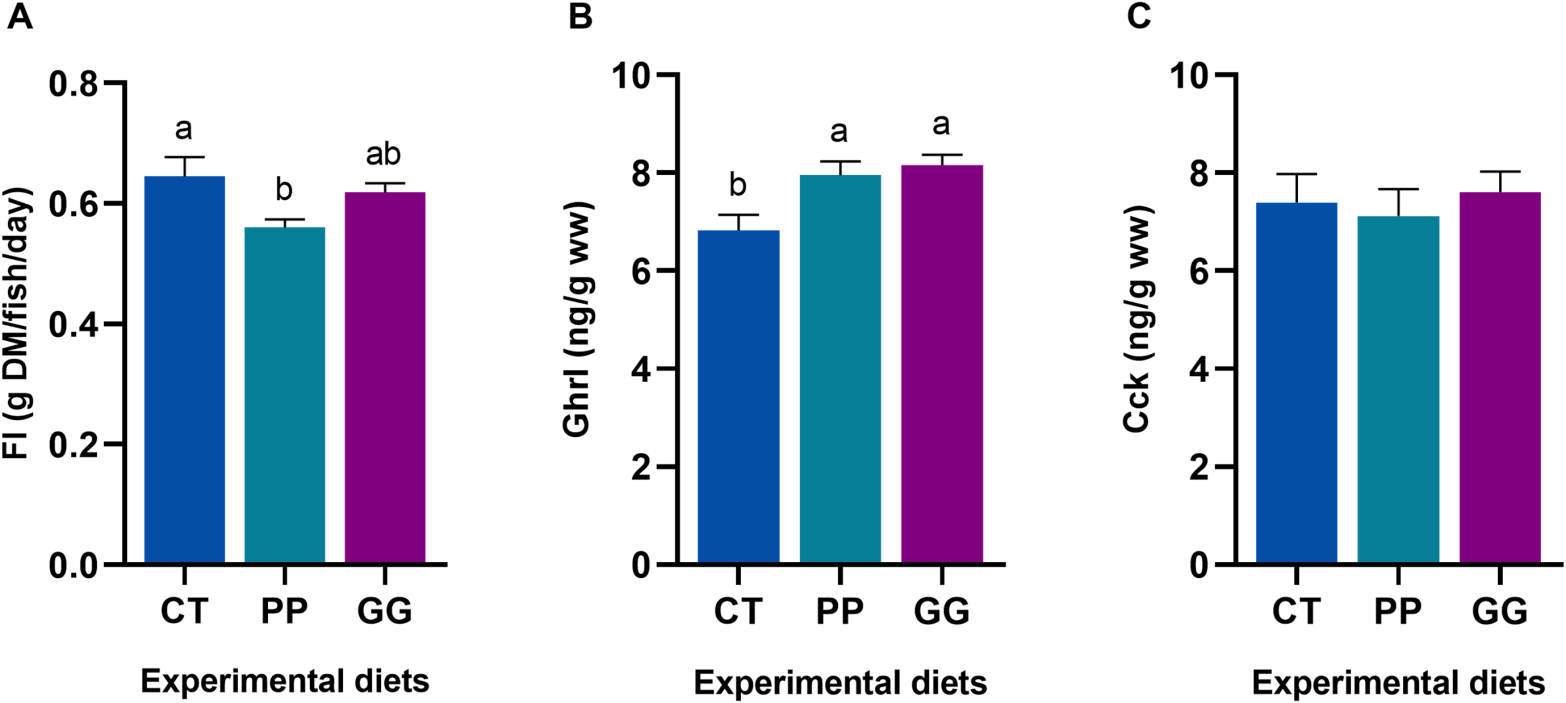
**(A)** Feed intake (FI) values per diet during the 92-day feeding period. Mean values of **(B)** ghrelin (Ghrl; GIT-S1) and **(C)** cholecystokinin (Cck; GIT-S2) over the first 24 h after the last feeding (09:00; average of Cf-2h, Cf-6h, and Cf-24h) in juvenile *Sparus aurata* fed to apparent visual satiety for 92 days with three experimental diets (CT: control diet based on 20% FM; PP: diet with 60% replacement of FM by hydrolyzed plant protein; GG: PP diet supplemented with 2% of the *LB-Green_Grape_*). Hormone concentrations are expressed as ng·g^-1^ wet weight. Data are presented as mean ± SEM (n = 18–21 fish randomly sampled across three replicate tanks). Feed intake is presented as mean ± SEM from triplicate tanks. Different letters indicate statistically significant differences among experimental diets, as determined by one-way ANOVA followed by Tukey’s *post hoc* test (*p < 0.05*).

FI, expressed as grams of dry matter consumed per fish per day (g DM/fish/day), was significantly higher in fish fed the CT diet and lowest in those fed the PP diet (*p < 0.05*; **Figure 8A**). The GG-fed group showed intermediate FI values, which did not differ significantly from the other two dietary treatments. Concentrations of Ghrl and Cck over the first 24 h post-feeding (calculated as the average of hormone levels at 2, 6, and 24 h) were compared among the three dietary groups. Ghrl levels were significantly higher in both high plant-based diets (PP and GG) relative to CT (*p < 0.05*; **Figure 8B**). In contrast, Cck values did not differ significantly between dietary treatments (*p > 0.05*; **Figure 8C**).

## 4 Discussion

Feeding regulation involves endocrine signals from both the central nervous system and peripheral organs, mainly the GIT, which act via the vagus nerve or by crossing the blood-brain barrier (5). Satiety is promoted by peptides such as CCK, while GHRL, mainly secreted by the stomach, stimulates appetite (8). This study quantified Ghrl and Cck protein levels along the GIT in juvenile *S. aurata*, evaluating their distribution, temporal variation, and dietary response, thus providing a functional perspective beyond gene expression.

### 4.1 Gastrointestinal distribution of Ghrl and Cck

Ghrl is a multifunctional hormone involved in growth regulation, FI, energy homeostasis, immunity, and gastrointestinal physiology (13, 31). In mammals, GHRL-producing cells are distributed throughout the GIT but are most abundant in the gastric body, the main source of circulating GHRL (32). In fish, mature Ghrl derives from preproghrelin and is expressed in several tissues. It is generally found in the stomach of gastric species (3) and in the intestine of agastric species (5, 33), reflecting high functional plasticity. Ghrl mRNA and protein have been detected in both stomach and intestine of fish (33–37). Most studies rely on gene expression rather than peptide quantification, which is an important distinction because mRNA-protein relationships vary with energy and stress status, as well as species (3). Ji et al. (38), for instance, found higher *ghrl* expression in the posterior intestine of *Megalobrama amblycephala*, suggesting species-specific tissue distribution. Few studies have simultaneously assessed the localization of Ghrl-producing cells and protein presence across the GIT. Using immunohistochemistry and Western blot, Arcamone et al. (39) reported high abundance in the stomach but sparse presence in the intestine of *Dicentrarchus labrax*, while Barrios et al. (30) found Ghrl immunoreactivity only in glandular intestine cells of *Prochilodus lineatus*.

It is important to note that the fish used in the initial characterization phase were not part of the nutritional challenge. They were maintained on a standard commercial diet, with the sole objective of mapping the baseline distribution of Ghrl and Cck levels along the GIT. Although Ghrl has previously been measured in blood plasma of *S. aurata* (24), this study reports, for the first time in this species, the hormone levels along the GIT itself. Using a Fish Ghrelin ELISA, we found that Ghrl concentrations peak in distal gut segments, with S5 (the second half of the posterior intestine, including the rectum) consistently exhibiting the highest levels under both basal and postprandial conditions. These findings suggest that the mid and distal intestine contribute substantially to Ghrl dynamics. These high levels are unlikely to result from potential cross-reactivity of the Ghrl kit with motilin, as the anterior intestine is considered the primary site of motilin synthesis (40). Therefore, this Ghrl pattern may occur due to local or systemic signaling related to digestion, nutrient absorption, or intestinal transit, shaped by species-specific physiology and energy demands. The markedly higher concentrations in S5 may indicate a local role in modulating motility and the terminal phase of digestion, as observed in teleosts (41) and other vertebrates (42). The observed pattern could also reflect local Ghrl storage within enteroendocrine cells or enteric nerve fibers, which may explain the high tissue levels even in the absence of major differences in mRNA expression reported for other species (3).

Cck is an anorexigenic peptide hormone that regulates digestive physiology by inhibiting gastric emptying, stimulating pancreatic secretion, and modulating intestinal motility (43, 44). In teleosts, its distribution is highly variable and species-specific, reflecting differences in anatomy and feeding strategies. This variability is also observed in immunohistochemical studies, which reveal a marked diversity in the localization of Cck-producing cells. In agastric species, these cells are dispersed throughout the intestine (45), whereas in gastric fish they usually concentrate in anterior and mid-intestinal segments (29, 30). In *Gadus morhua*, they are abundant in the stomach and proximal intestine near the pyloric caeca, decreasing distally (29). In *Diplodus sargus* adults, they are enriched in the anterior mid-intestine and gradually decline posteriorly (46). Conversely, in *Oligosarcus hepsetus*, Cck cells are confined to the posterior intestine, where they regulate motility (44). Molecular data confirm widespread intestinal expression, as in rainbow trout (*Oncorhynchus mykiss*) (47), although immunoreactivity is generally highest near the pyloric caeca (48).

Ontogenetic studies also show dynamic shifts in the distribution of Cck-producing cells along the GIT. In *Diplodus puntazzo* larvae, Cck cells first appear in mid- and posterior intestine, later concentrating anteriorly, with posterior abundance increasing alongside gastric gland development (46). Similarly, in larvae of *Paralichthys olivaceus*, *Thunnus thynnus*, and *Plecoglossus altivelis*, Cck cells localize to the mid-anterior intestine or pyloric caeca depending on developmental stage (49–51).

In our study, the highest Cck levels were detected in S5 (rectal region). This is consistent with previous findings in *S. senegalensis* (27) and with elevated *cck-l* expression in the pyloric caeca and posterior intestine of *Salmo salar* (52). These elevated levels are probably not due to cross-reactivity of the Cck kit with gastrin, since the stomach is considered the main site of gastrin synthesis in fish possessing a true stomach (53). Such posterior enrichment in *S. aurata* suggests a role for Cck beyond early digestion, including regulation of water absorption, content mixing, and defecation-related motility (54). Distal Cck has further been linked to feedback regulation of digestion and potential anti-inflammatory functions (16, 52), which may explain the consistently high S5 levels. When hormone concentrations were integrated over the first 24 h after feeding (2, 6, and 24 h) mean Cck levels peaked in S2 (anterior intestine with pyloric caeca), consistent with most previous studies (29, 30, 46). The discrepancy between the elevated Cck observed in S5 at the baseline characterization (4 h) and the S2 dominance in the integrated analysis likely reflects differences in sampling design and metrics (single time point vs. 24 h average) but may also point to region-specific temporal dynamics in Cck synthesis, storage, or release along the intestine.

### 4.2 Postprandial and fasting responses of appetite hormone levels

Ghrl and Cck are pivotal in appetite regulation, but their concentrations fluctuate in response to changes in feeding status, fasting duration, and environmental factors such as temperature and stress (3, 52). Spatial characterization provides a baseline distribution yet does not capture the temporal variability essential to their function.

In mammals, circulating GHRL rises during fasting as gastric cells release stored hormone, and declines after refeeding (32). The increase during fasting and the preprandial peak have been observed in humans and rodents, supporting the conserved orexigenic function of circulating GHRL (55, 56). Similar fasting-induced responses have been reported in several teleost species, with increased intestinal and brain *ghrl* expression and elevated circulating levels (5, 12, 15, 33, 36–38, 57, 58). For example, in *Carassius auratus*, Unniappan et al. (57) demonstrated coordinated increases in preproghrelin mRNA in the pituitary and intestine together with higher serum Ghrl levels during fasting, indicating that Ghrl synthesis and secretion can be tightly coupled in teleosts. This response is considered an adaptive mechanism that stimulates appetite and conserves energy during food deprivation (59). However, interspecific variability exists (3). In *O. mykiss*, for example, plasma Ghrl levels decrease during fasting, indicating that in this species Ghrl may relate more to metabolism and growth than to short-term appetite (59).

Our results in *S. aurata* are consistent with the established orexigenic role of Ghrl in fish. In the stomach (S1), Ghrl levels declined markedly after prolonged fasting (24 h and 7 days), a pattern that may reflect mobilization of the hormone from gastrointestinal stores. Although plasma levels were not measured, this interpretation aligns with observations in mammals (32) and teleosts (24, 57), reporting increased circulating (GHRL) after fasting. However, tissue levels represent peptide inventory rather than direct secretion.

Divergent temporal variations in Ghrl and Cck values in response to fasting were observed between segments. In the proximal anterior intestine (S2), Ghrl levels rebounded after seven days of fasting. Such findings suggest segment-specific regulation of Ghrl (37) and a potential local accumulation of the hormone in S2 during prolonged fasting. This is consistent with evidence that circulating GHRL reflects overall energy balance, not just individual meals, in mammals (55) and fish (60).

In S5 (which includes the second half of the posterior intestine and the rectum), Ghrl levels were generally higher than in S1 (stomach) and S2 (proximal section of the intestine). Although they began to decline a few hours after feeding, Ghrl levels remained relatively stable, suggesting a local role in the posterior intestine, potentially modulating motility and terminal digestive processes, as has been observed in mammals (42) and fish (41). Experimental evidence in zebrafish (*Danio rerio*) suggests that Ghrl stimulates intestinal contractions. This effect may involve direct actions on smooth muscle or indirect pathways through the enteric nervous system, indicating that both local production and Ghrl from extra-intestinal sources contribute to gut motility regulation (41). In our study, higher levels of Ghrl in S5 shortly after feeding may indicate a significant facilitation of evacuation, whereas lower levels during prolonged fasting could reflect the absence of intestinal content. Mammalian studies also show that circulating GHRL can decrease by ~50% under normal gastric emptying (32), supporting its role in coordinating final digestion and gut evacuation. In this context, the increased tissue levels may reflect the buildup of a functional reserve rather than a mere reduction in hormone release.

CCK is secreted into the bloodstream after the ingestion of protein and fat, regulating food consumption, digestion, and nutrient absorption (61). In fish, feeding typically enhances gene expression or tissue levels of this hormone (16, 18, 62–66). Our results may reflect a balance between potential postprandial release and retention in the intestinal epithelium during fasting. Specifically, the lower Cck levels observed a few hours after feeding could be consistent with secretion from the intestinal epithelium into the bloodstream to trigger satiety (61), followed by tissue accumulation during fasting, although this remains a hypothesis, given that plasma Cck was not measured. Similar patterns occur in larvae of different fish species, where intestinal Cck is low when the gut is full and increases as it empties (67, 68), supporting the interpretation that feeding drives release and resynthesis rather than a circadian effect. Furthermore, in *O. mykiss*, postprandial Cck plasma levels peak approximately 4 h after feeding and persist beyond 6 h (69), supporting secretion from the GIT into the circulation. In contrast, the rise in GIT hormone levels during fasting may serve as a preparatory storage mechanism in anticipation of feeding.

As with Ghrl, Cck patterns varied across the GIT segments, indicating differential regulation at the segment level (2, 27). In the stomach (S1), Cck increased during feed restriction, but the changes (magnitude) were less pronounced than in S2 and S5. In the anterior intestine containing pyloric caeca (S2), which harbors the highest density of Cck-producing cells (29, 30), previous studies have reported rapid postprandial increases in *cck* mRNA expression followed by declines during fasting (25, 38, 62, 63, 66). In line with these findings, we observed a progressive accumulation of Cck during prolonged fasting, peaking after seven days. This may reflect local storage acting as a reservoir for rapid activation of satiety signaling and digestive processes once feeding resumes (27).

In the posterior intestine (S5), where baseline Cck levels were highest, tissue concentrations enhanced noticeably only after 24 h and 7 days of fasting. Although similar to S1, S5 exhibited the largest fluctuations, from very low levels shortly after feeding to markedly higher values during prolonged fasting. These results reiterate transcriptional findings in *D. sargus*, where fasting reduced *cck2* mRNA expression throughout the intestine (16), suggesting a complex role, possibly mediated by isoform-specific regulation. The delayed and more pronounced increase in the distal intestine compared with S2 may reflect a differential function in this segment during prolonged fasting, contributing to distal motility, water absorption, and intestinal waste elimination rather than immediate satiety signaling (54).

### 4.3 Dietary modulation of appetite-regulatory hormones (Ghrl and Cck) under a fasting-refeeding challenge

Dietary control is a practical tool in aquaculture; however, little is known about how key appetite-regulating hormones, such as Ghrl and Cck, respond to changes in diet composition. Most studies have focused on feeding–fasting transitions, leaving the role of macronutrient profiles largely unexplored.

Different factors, such as energy status and food composition, can influence Ghrl synthesis and secretion (3, 9, 59). In mammals, circulating GHRL declines rapidly upon nutrient absorption, especially glucose, lipids, and amino acids (32). During early digestion (2–6 h), gastric Ghrl levels remained relatively high across all diets, reflecting ongoing local synthesis and the fact that postprandial suppression does not immediately deplete tissue stores, with only minor effects of diet formulation (12, 24, 55). By 24 h, however, fish fed the control diet (CT) showed a marked reduction in gastric Ghrl compared with earlier time points and the high plant protein diets (PP and GG). This decrease is likely due to greater peptide release into circulation, potentially signaling the return of appetite. In contrast, persistently high gastric Ghrl in PP and GG diets suggests attenuated postprandial secretion or compensatory local synthesis under lower nutrient digestibility (26). These differences between dietary groups align with evidence that heavier meals suppress GHRL more than lighter meals (70) and mirror caloric effects on appetite in mammals (71). After refeeding, gastric Ghrl content rose shortly and then tended to decrease by 24 h in fish fed all dietary groups, consistent with possible secretion of this hormone into circulation and the potential appearance of hunger signs. In this sense, in humans, gastric GHRL acts as a physiological initiator of feeding, with the stomach positioned to sense short-term energy changes (55).

In *S. aurata*, chronic exposure to less digestible diets can trigger compensatory hyperphagia and local *ghrl* upregulation (26). However, in our study, FI was highest in CT diet, lower in PP diet, and intermediate in GG diet, while gastric Ghrl remained elevated in fish fed high plant-based diets (PP/GG) during the first 24 h (average across 2, 6, and 24 h). These patterns may indicate that, rather than enhancing orexigenic drive, an attenuated postprandial release of Ghrl into circulation could have contributed to a weaker hunger signal, an interpretation that aligns with the lower FI observed in these groups and its partial recovery with the inclusion of the functional additive.

Despite these patterns, an additional methodological consideration must be acknowledged. The use of a visual-satiety feeding protocol, standard in aquaculture but inherently variable in intake, raises the question of whether the lower FI observed in fish fed the plant-based diet reflects sensory rejection of the diet (72, 73) or an intrinsic modulation of appetite-regulatory pathways driven by its nutritional composition. In our dataset, however, the temporal pattern of gastric Ghrl in PP-fed fish does not align with what would be expected from a simple orexigenic response to reduced intake. Instead, Ghrl remained elevated during the first 24 h post-feeding, suggesting attenuated postprandial release (hypothetical secretion into plasma) or local compensatory synthesis. Additionally, we observed a pronounced postprandial decrease in Cck at 6 h after feeding in PP-fed fish, which may reflect either enhanced secretion into the plasma, potentially prolonging the satiety state, or a local reduction in its synthesis.

However, this response could also be a consequence of lower FI, as meal size could modulate Ghrl secretion into the circulation and, therefore, hunger-satiety signals. Previous observations in *O. mykiss* showed that postprandial Cck and Ghrl secretion can be influenced by diet composition, rather than meal size *per se*, particularly by differences in lipid:protein ratios (59, 69). Furthermore, the GG group showed a partial recovery of FI, despite sharing the same plant-protein base as PP and differing only by the 2% microalgae-grape feed additive (the diets being isonitrogenous and isolipidic). This supports the idea that specific dietary components can modulate Ghrl and Cck interplay beyond voluntary consumption alone. Grape by-products and their polyphenols can beneficially influence growth performance, gut integrity, antioxidant status, and immune function in fish (74), consistent with the enhanced physiological responses observed in the GG group.

Consistent with the FI patterns, growth performance and feed utilization metrics provide essential context for interpreting the endocrine responses. Diets rich in plant-protein ingredients resulted in reduced growth and lower feed efficiency, as indicated by decreased WG, SGR, and elevated FCR, reflecting limitations in nutrient utilization and potential anti-nutritional effects. In contrast, the inclusion of the functional feed additive in the GG diet partially mitigated these effects, as previously observed in gilthead seabream supplemented with microalgae-based functional additives (72, 75). Taken together, these results leave unresolved whether the reduced FI reflects palatability constraints or whether the plant-based formulation actively modulates postprandial Ghrl-Cck dynamics. Thus, while hormonal profiles appear primarily influenced by diet composition, the potential role of caloric restriction cannot be fully excluded. It will be essential to disentangle sensory effects from true endocrine modulation. Nonetheless, designs that control intake across dietary groups would be valuable for more definitively disentangling these interacting factors in future work.

Beyond appetite, GHRL also modulates mucosal integrity and inflammation. In humans, endogenous GHRL rises in response to mucosal injury and inflammatory bowel disease, promoting epithelial proliferation, barrier maintenance, and anti-inflammatory effects via vagal pathways (76). Although intestinal pathology was not assessed, higher gastric Ghrl in fish fed PP and GG diets may reflect, in addition to orexigenic adjustments, mucosal stress due to lower digestibility or antinutritional factors present in these diets (even with enzymatic treatment). This fact has been previously suggested by our Research Group, based on electrophysiological and histological evidence of mucosal stress in *S. aurata* specimens fed plant-based diets (72).

Several studies have shown that proteins and lipids are the strongest stimuli for Cck release in fish, whereas carbohydrates elicit weaker or transient responses. In *Seriola quinqueradiata*, casein, oleic acid, or triolein increased intestinal *cck* mRNA, while starch had minimal effect (77). Fishmeal also stimulates Cck synthesis in the intestine and pyloric caeca, highlighting the modulatory role of dietary protein (20). Specific amino acids or proteins enhance Cck levels in larvae of *Clupea harengus* (78) and *S. aurata* (79), and high-protein/low-carbohydrate diets stimulate Cck expression in *S. aurata* adipose tissue (25). Consistently, studies in *O. mykiss* have shown that postprandial Cck secretion is modulated by the dietary lipid:protein ratio, with higher circulating Cck levels detected in fish fed lipid-rich diets, reinforcing the notion that dietary lipids also act as potent stimuli of hormone release (69). Mammalian studies support this pattern, with proteins, amino acids, and lipids as primary CCK drivers and carbohydrates inducing weaker responses (80–82). This nutritional context may explain the slightly higher, though non-significant, anterior intestine Cck levels detected in fish fed the CT diet.

A striking feature across diets was the pronounced Cck peak at 24 h post-refeeding (Rf-24h) in the anterior intestine, absent at 24 h after continuous feeding (Cf-24h). After prolonged fasting, a single meal may trigger increased synthesis and storage for rapid release upon subsequent meals (27). This pattern aligns with the conserved tissue distribution of Cck receptors in teleosts, where *cck-1* receptors are widely expressed along the GIT, mediating local effects on digestion and appetite (10). Supporting this, *in vivo* studies with *S. quinqueradiata* have shown that *cck-1r* mRNA levels increase in the gallbladder and pyloric caeca following feeding, and *in vitro*, addition of Cck to cultured pyloric caeca tissue enhances expression of both *cck-1r* and digestive enzymes (66). Together, these observations suggest that the post-refeeding Cck surge in the anterior intestine may directly facilitate digestive processes, including enzyme secretion and intestinal motility. In this context, the expression of digestive proenzymes and their activity have been shown to follow significant circadian rhythms in *S. aurata* larvae and juveniles (23, 83). The authors attributed elevated proenzymes levels either to an anticipatory response before feeding, enhancing nutrient utilization, or to the presence of feed within the corresponding GIT segment when measuring enzyme activity. Consistently, in *S. senegalensis*, a single daily feeding induced a marked circadian rhythm in intestinal Cck, with Cck peaks followed by increases in trypsin and chymotrypsin activity, the main proteolytic enzymes in this species (27). These findings align with the anticipatory interpretation of the Cck peak observed here. They also parallel mammalian physiology, where rapid, meal-related satiety relies on short-acting intestinal signals such as CCK and gastric distension, rather than delayed GHRL suppression (70). Thus, the Rf-24h Cck peak may reflect a dual functional profile, combining its well-established role as a rapid, meal-related satiety signal with a slower, anticipatory component that supports digestive preparedness by enhancing local processes such as enzyme secretion and intestinal motility. Further experiments would be necessary to confirm this functional interpretation.

The interaction between Cck and Ghrl is notable. Functional antagonism occurs in both mammals and fish, including *S. aurata* (23, 38, 52, 84, 85). In our study, Cck accumulated in the GIT as the postprandial period progressed, which may reflect reduced release from the intestinal epithelium. In contrast, tissue Ghrl levels showed a pattern consistent with increased orexigenic drive, although plasma concentrations were not measured. This inverse relationship also appeared across diets and during the refeeding state, with a significant negative correlation between stomach (S1) Ghrl and anterior intestine (S2) Cck levels. These findings highlight their complementary roles in energy homeostasis and the spatiotemporal coordination of feeding in *S. aurata*. Notably, the correlation between Ghrl and Cck during the fasting state was not statistically significant, likely due to the low number of samples available, which may have limited the statistical power to detect existing associations. Interestingly, the diet enriched in plant-based ingredients and supplemented with the functional additive exhibited a distinct postprandial pattern, with a significant positive correlation, suggesting that the additive may influence the balance between Ghrl and Cck after feeding. Although the underlying mechanisms are not yet clear, such effects could partially contribute to the improved FI observed in this group and justify further, more detailed studies on functional supplementation and the physiological impacts of plant-rich diets.

## 5 Conclusions

This study expands current knowledge of appetite regulation in *S. aurata* by providing, for the first time, a detailed quantification of Ghrl and Cck proteins along the GIT. The results reveal marked spatial differences, with both hormones accumulating at high levels in the posterior intestine (S5), suggesting an additional role in modulating distal motility and terminal digestive processes beyond their classical functions in hunger and satiety. Temporal analyses across postprandial, fasting, and refeeding conditions highlighted dynamic, segment-specific adjustments, with Ghrl and Cck showing complementary, inverse fluctuations in most cases, consistent with their orexigenic and anorexigenic roles. Diet composition further influenced these responses. Both the PP and GG diets maintained elevated gastric Ghrl (S1) levels while decreasing intestinal Cck (S2) post-feeding, particularly in the PP diet. Such a pattern may reflect prolonged satiety and reduced FI, or changes in hormone release associated with lower caloric intake, with the PP diet reducing growth and feed efficiency, effects partially mitigated by the functional additive. Additionally, the pronounced Cck surge observed after refeeding following prolonged fasting illustrates the adaptive plasticity of this regulatory system, reflecting anticipatory preparation for renewed FI and digestive activity. Taken together, these findings underscore how appetite control in *S. aurata* emerges from a finely tuned spatiotemporal interplay between hormones (Ghrl and Cck) and diet, nutritional status, with parallels to mammalian systems. This integrative perspective provides valuable insights for improving feeding strategies, growth performance, and welfare in aquaculture.

## 6 Future perspectives

Future studies should broaden the endocrine scope beyond Ghrl and Cck to encompass additional appetite-related peptides acting both peripherally and centrally, thereby capturing a more integrated hormonal landscape underlying feeding regulation in *S. aurata*. Particular attention should be directed to intermediate and distal intestinal regions, where marked Ghrl and Cck accumulation was detected in the present work. Assessing whether this reflects segment-specific functional roles, altered trafficking of peptide stores, or dynamic redistribution along the digestive tract will be key to refining our understanding of gut-derived appetite cues. A complementary priority will be the integration of gastrointestinal measurements with circulating hormone levels, enabling distinction between tissue inventories and true secretory events and allowing local endocrine patterns to be linked with systemic responses. Parallel evaluation of gene expression (e.g., *ghrl*, *cck*, receptor pathways) would further clarify whether observed changes arise from modulation of synthesis, release, or degradation, among others.

## Data Availability

The original contributions presented in the study are included in the article/**Supplementary Material**. Further inquiries can be directed to the corresponding authors.
