# Supplementary material for: Spatiotemporal characterization of ghrelin and cholecystokinin levels in the gastrointestinal tract of juvenile Sparus aurata: effects of feeding status and diet composition
Source: Front Endocrinol (Lausanne). 2026 Jan 27;17:1734169. doi: 10.3389/fendo.2026.1734169 (PMC12886005; doi:10.3389/fendo.2026.1734169)
Supplement: Supplementary file 1 [file SupplementaryFile1.pdf]

# Supplementary Material

## 1. Theoretical validation of Cck and Ghrl ELISA kits for *Sparus aurata*

*In silico* evaluation of antibody-antigen compatibility was performed to assess the applicability of the commercial ELISA kits for measuring cholecystokinin (Cck) (Fish Cholecystokinin-8 ELISA Kit; Ref. No. MBS069488; MyBioSource, USA) and ghrelin (Ghrl) (Fish Ghrelin ELISA Kit; Ref. No. MBS1601713; MyBioSource, USA) in gilthead seabream (*Sparus aurata*) gastrointestinal tract.

### 1.1. Cck

In *S. aurata*, two complete Cck deduced amino acid (aa) sequences are available on GenBank: XP_030268255.1 (UniProt ID A0A671UND2) and XP_030250950.1 (UniProt ID A0A671WWZ7), belonging to genes *cckb* and *ccka*, respectively.

The Cck ELISA kit used in this study was developed based on the Japanese flounder (*Paralichthys olivaceus*) Cck sequence (UniProt ID O57312). For *S. aurata*, the monoclonal capture antibody epitope (aa 26-75) showed 80% aa identity (40/50 aa) and 86% similarity (43/50 aa) with the predicted Cck sequence XP_030268255.1, whereas homology with XP_030250950.1 was negligible. In contrast, the polyclonal detection antibody epitope (aa 116-123) was 100% identical (8/8 aa) to both *S. aurata* Cck sequences (Figure S1). Taken together, these data indicate that the ELISA kit most likely detects Cck-like immunoreactivity predominantly derived from the *cckb* gene in the gastrointestinal tract of *S. aurata*.

Moreover, sequence analysis revealed partial overlap between the detection antibody epitope and the predicted gastrin domain in *S. aurata* (aa 121-134 in XP_030268255.1 and 24-62 in XP_030250950.1) (Figure S1), with five shared aa, indicating that cross-reactivity with gastrin-related peptides cannot be fully excluded.

```
XP_030250950.1   MNVGICVCVLLAALSSRSLSLPSHTVS--QRAVGEALP--------SDSLPPHHTRQARS  50
XP_030268255.1   MAAGLCVCVVLAVLCTSCLGLPFSSQLLDEGQRSAAVPSEALLEADTHSLGEHHLQHSRS  60
O57312           MTAGLCVCVLLAVLCTSCLGHPISSQHLDEGQRSISTPSEALLEADTHSLGEPHLRQSRS  60
                 * .*:****:**.*.: .*. *  :    :   . : *        :.**   * :::**

XP_030250950.1   APALPSGQLASYTQPQEGADARSSLSQLLARLISRKGSPYQTRSSLTSRASGLAPSHRIK  110
XP_030268255.1   APQL-----KALPPAEEDADSRANLSELLARLISSRKGSVRRNSTANSRGGGLSANHRIA  115
O57312           APQL-----KSLPVAEEDGDSRANLSELLARLISSRKGSVRRNSTAYS--KGLSPNHRIA  113
                 ** *      :    :*..*:*:.**:******* : .  : .*:  *    **: .***

XP_030250950.1   DRDYLGWMDFGRRSAEEYEYSS  132
XP_030268255.1   DRDYLGWMDFGRRSAEEYEYSS  137
O57312           DRDYLGWMDFGRRSAEEYEYSS  135
                 **********************
```

**Figure S1.** Alignment of the *P. olivaceus* Cck aa sequence (O57312), which was used to generate the immunogens for the commercial ELISA kit, with the two predicted *S. aurata* Cck aa sequences (XP_030268255.1 and XP_030250950.1). Regions shown in bold and bold + underlined correspond to the epitopes of the capture and detection antibodies, respectively. The region enclosed within the box indicates the predicted gastrin domain.a

### 1.2. Ghrl

In *S. aurata*, one complete deduced ghrelin (Ghrl) aa sequence is available in GenBank (XP_030274358.1; UniProt ID A0A2Z4M046).

The Ghrl ELISA kit used in this study was developed based on the rainbow trout (*Oncorhynchus mykiss*) Ghrl sequence (UniProt ID Q76IQ4). For *S. aurata*, the monoclonal capture antibody epitope (aa 68-111) showed 61% aa identity (27/44 aa) and 81% similarity (36/44 aa) with the predicted Ghrl sequence. In contrast, the polyclonal detection antibody epitope (aa 41-111) showed 51% identity (37/73 aa) and 68% similarity (50/73 aa) to the *S. aurata* Ghrl sequence (Figure S2). Although lower sequence identity may reduce antibody binding efficiency, the assay can still be used to compare relative differences in Ghrl-like immunoreactivity among gastrointestinal segments in *S. aurata*, as performed in the current study.

Moreover, sequence analysis revealed strong overlap between the detection antibody epitope and the predicted motilin domain in *S. aurata* (aa 59–97), with 39 identical aa (Figure S2), indicating a substantial likelihood of cross-reactivity with motilin-like peptides.

```
XP_030274358.1      MFLKRNTCLLVFLFCSLTLWCKSTSAGSSFLSPSQKPQNR---GKSSRVGRQVMQ-----  52
Q76IQ4              MPLKRNTGLMILMLCTLALWAKSVSAGSSFLSPSQKPQVRQGKGKPPRVGRRDIESFAEL  60
                    * ***** *:::::*:*:**.**.************** *   **  ****: ::

XP_030274358.1      -EPHQPTDDKHITISAPFEIGISMTEEDYAEYGVVLQEIIQRLLGGTEAAEGPPQL  107
Q76IQ4              FEGPLHQEDKHNTIKAPFEMGITMSEEEFQEYGAVLQKILQDVLGDTATAE-----  111
                     *     :*** **.****:**:*:**:: ***.***:*:* :**.* :**
```

**Figure S2.** Alignment of the *O. mykiss* ghrelin (Ghrl) aa (Q76IQ4), which was used to generate the immunogens for the commercial ELISA kit, with the predicted *S. aurata* Ghrl aa sequence (XP_030274358.1). Regions shown in bold + grey and bold + underlined correspond to the epitopes of the capture and detection antibodies, respectively. The region enclosed within the box indicates the predicted motilin domain.

## 2. Example standard curves with *Sparus aurata* Cck and Ghrl measurements

To illustrate the standard curves generated using the commercial ELISA kits and the relative positioning of *S. aurata* sample measurements, Figure S3 presents representative data from all gastrointestinal tract samples obtained in Phase 1. This phase was selected because a wider range of hormone concentrations was expected across the different gut segments. For samples analyzed in Phases 2 and 3 (data not shown), absorbance values also consistently fell within the dynamic range of the standard curves and fitted the curves well, avoiding upper or lower extremes where extrapolation could compromise reliability.

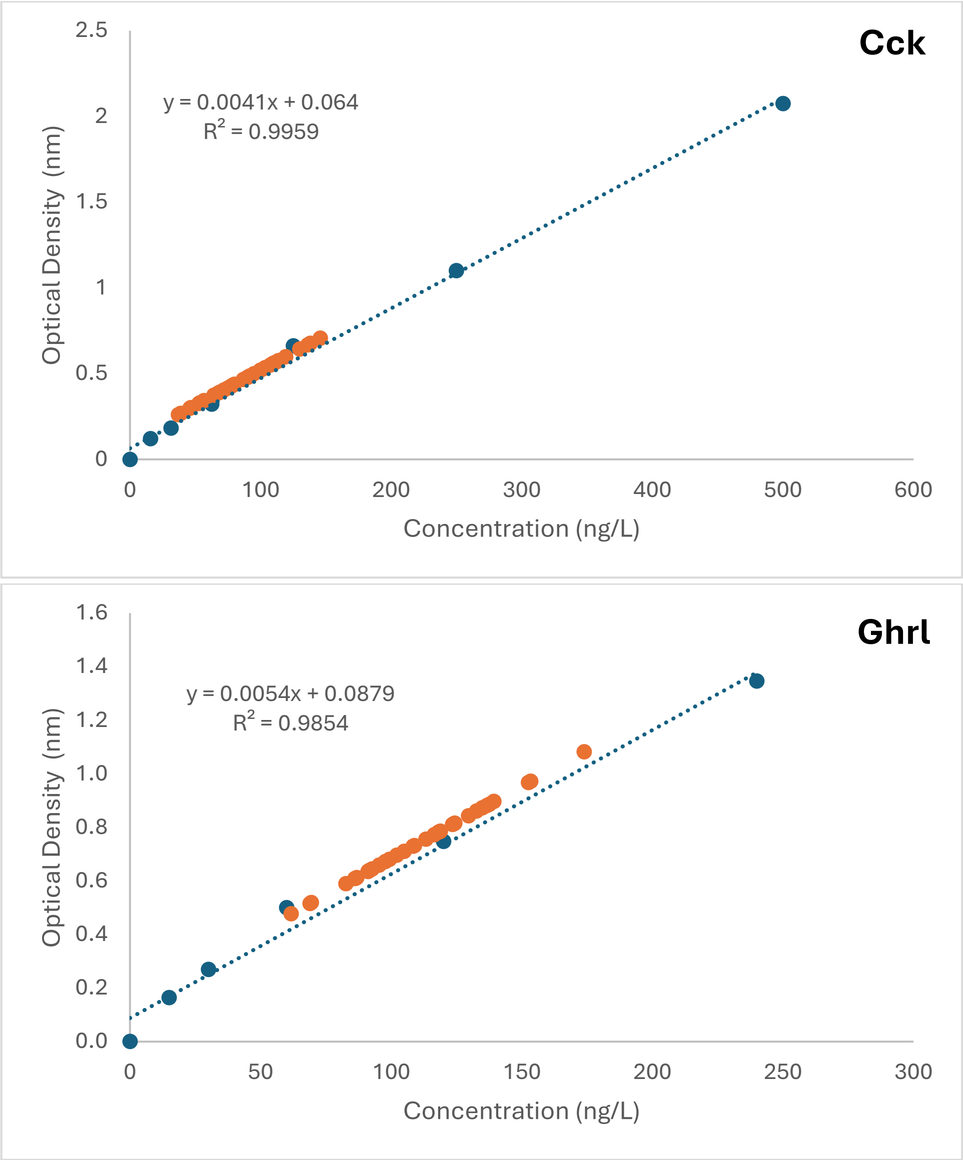


**Figure S3.** Standard curves for the hormone measurement assays. Blue points represent the kit standards (for Cck: blank, 15.6, 31.25, 62.5, 125, 250, and 500 ng/L; for Ghrl: blank, 15, 30, 60, 120, 240 ng/L), and orange points represent hormone measurements from gastrointestinal tract samples of *S. aurata* from Phase 1 of the experimental design in the current study.
